# Supplementary material for: Isolation and Characterization of Antibacterial Carotane Sesquiterpenes from Artemisia argyi Associated Endophytic Trichoderma virens QA-8
Source: Antibiotics (Basel). 2021 Feb 20;10(2):213. doi: 10.3390/antibiotics10020213 (PMC7924333; doi:10.3390/antibiotics10020213)

## Supplementary Material

# Sesquiterpenes from *Artemisia argyi* Associated Endophytic *Trichoderma virens* QA-8

**Xiao-Shan Shi [1,§], Yin-Ping Song [2,§], Ling-Hong Meng [1], Sui-Qun Yang [1], Dun-Jia Wang [3], Xing-Wang Zhou [3], Nai-Yun Ji [2,4], Bin-Gui Wang [1,4,*] and Xiao-Ming Li [1,4,*]**

[1] Key Laboratory of Experimental Marine Biology, Institute of Oceanology, Chinese Academy of Sciences, and Laboratory of Marine Biology and Biotechnology, Qingdao National Laboratory for Marine Science and Technology, Nanhai Road 7, Qingdao 266071, People's Republic of China; Shixs@qdio.ac.cn (X.S.S.); menglh@ms.qdio.ac.cn (L.H.M.); yangsuiqun@qdio.ac.cn (S.Q.Y.)

[2] Yantai Institute of Coastal Zone Research, Chinese Academy of Sciences, Yantai 264003, People's Republic of China; ypsong@yic.ac.cn (Y.P.S.); nyji@yic.ac.cn (N.Y.J.)

[3] College of Chemistry and Chemical Engineering, Hubei Normal University, Cihu Road 11, Huangshi 435002, People's Republic of China; dunjiawang@hbnu.edu.cn (D.J.W.); zhouxw@hbnu.edu.cn (X.W.Z.)

[4] Center for Ocean Mega-Science, Chinese Academy of Sciences, Nanhai Road 7, Qingdao 266071, People's Republic of China;

[§] These authors contributed equally to this work.

* Correspondence: wangbg@ms.qdio.ac.cn (B.G.W.); lixmqd@qdio.ac.cn (X.M.L.)

**Table of Contents**

Figure S1. $^{1}H$ NMR (500 MHz, DMSO-$d_6$) spectrum of compound **1**;
Figure S2. $^{13}C$ NMR (125 MHz, DMSO-$d_6$) and DEPT spectra of compound **1**;
Figure S3. COSY spectrum of compound **1**;
Figure S4. HSQC spectrum of compound **1**;
Figure S5. HMBC spectrum of compound **1**;
Figure S6. NOESY spectrum of compound **1**;
Figure S7. HRESIMS spectrum of compound **1**;
Figure S8.$^{1}H$ NMR (500 MHz, DMSO-$d_6$) spectrum of compound **2**;
Figure S9. $^{13}C$ NMR (125 MHz, DMSO-$d_6$) and DEPT spectra of compound **2**;
Figure S10. COSY spectrum of compound **2**;
Figure S11. HSQC spectrum of compound **2**;
Figure S12. HMBC spectrum of compound **2**;
Figure S13. NOESY spectrum of compound **2**;
Figure S14. HRESIMS spectrum of compound **2**;
Figure S15. $^{1}H$ NMR (500 MHz, DMSO-$d_6$) spectrum of compound **3**;
Figure S16. $^{13}C$ NMR (125 MHz, DMSO-$d_6$) and DEPT spectra of compound **3**;
Figure S17. $^{1}H$ -$^{1}H$ COSY spectrum of compound **3**;
Figure S18. HSQC spectrum of compound **3**;
Figure S19. HMBC spectrum of compound **3**;
Figure S20. NOESY spectrum of compound **3**;
Figure S21. HRESIMS spectrum of compound **3**;
Figure S22. $^{1}H$ NMR (500 MHz, DMSO-$d_6$) spectrum of compound **4**;
Figure S23. $^{13}C$ NMR (125 MHz, DMSO-$d_6$) and DEPT spectra of compound **4**;
Figure S24. $^{1}H$ -$^{1}H$ COSY spectrum of compound **4**;
Figure S25. HSQC spectrum of compound **4**;
Figure S26. HMBC spectrum of compound **4**;
Figure S27. NOESY spectrum of compound **4**;
Figure S28. HRESIMS spectrum of compound **4**;
Figure S29. $^{1}H$ NMR (500 MHz, DMSO-$d_6$) spectrum of compound **5**;
Figure S30. $^{13}C$ NMR (125 MHz, DMSO-$d_6$) and DEPT spectra of compound **5**;
Figure S31. $^{1}H$ -$^{1}H$ COSY spectrum of compound **5**;
Figure S32. HSQC spectrum of compound **5**;
Figure S33. HMBC spectrum of compound **5**;
Figure S34. NOESY spectrum of compound **5**;
Figure S35. HRESIMS spectrum of compound **5**;
Figure S36. Crystal packing of compound **1** at 293(2) K;
Figure S37. Crystal packing of compound **9** at 293(2) K;
Figure S38. Crystal packing of compound **10** at 293(2) K.

Figure S1. $^{1}$H NMR (500 MHz, DMSO-$d_6$) spectrum of compound **1**;

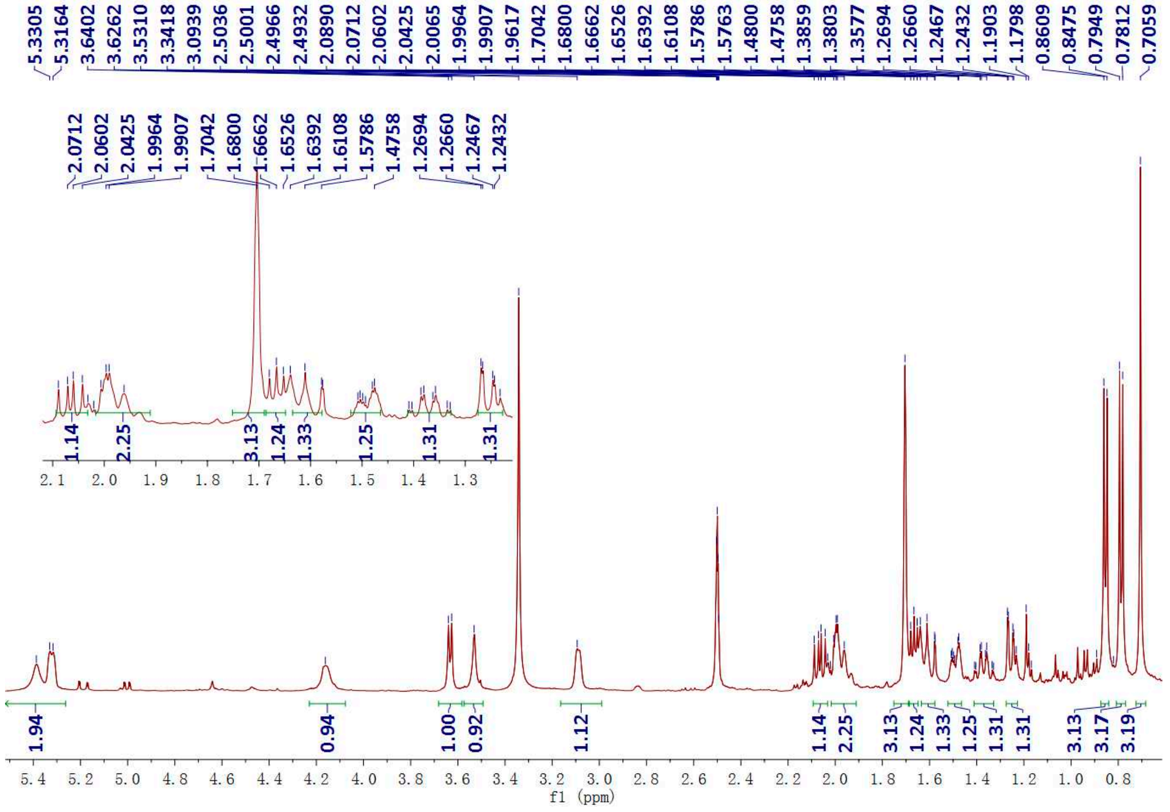


Figure S2. $^{13}$C NMR (125 MHz, DMSO-$d_6$) and DEPT spectra of compound **1**;

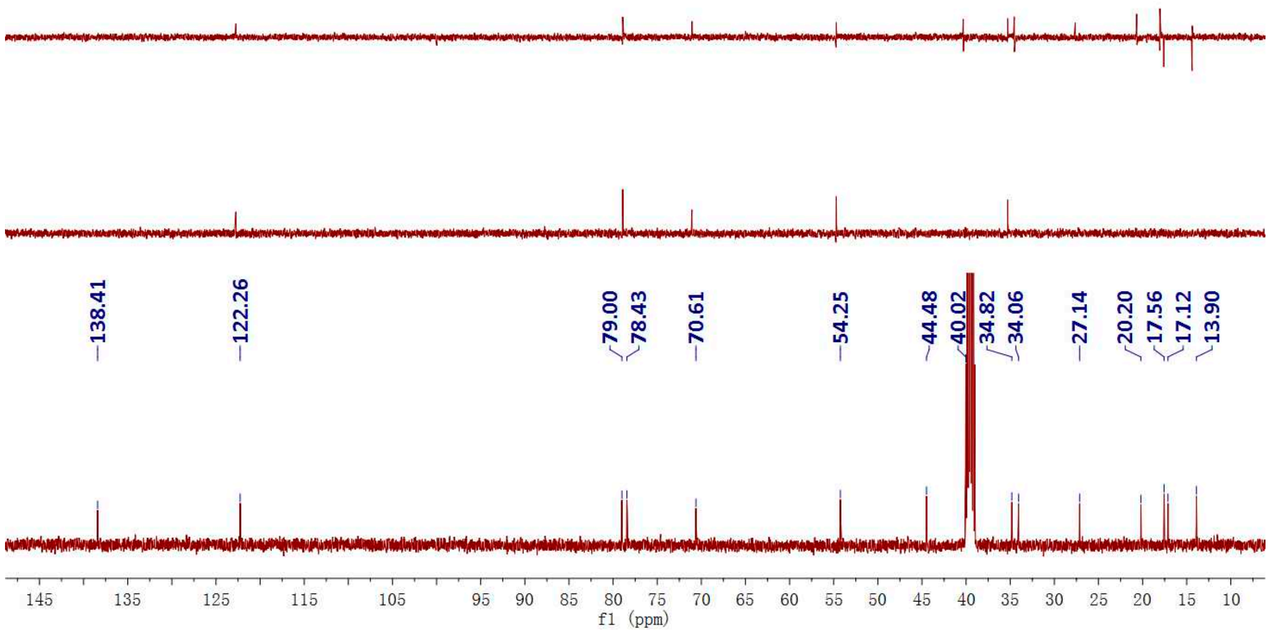

Figure S3. COSY spectrum of compound **1**;

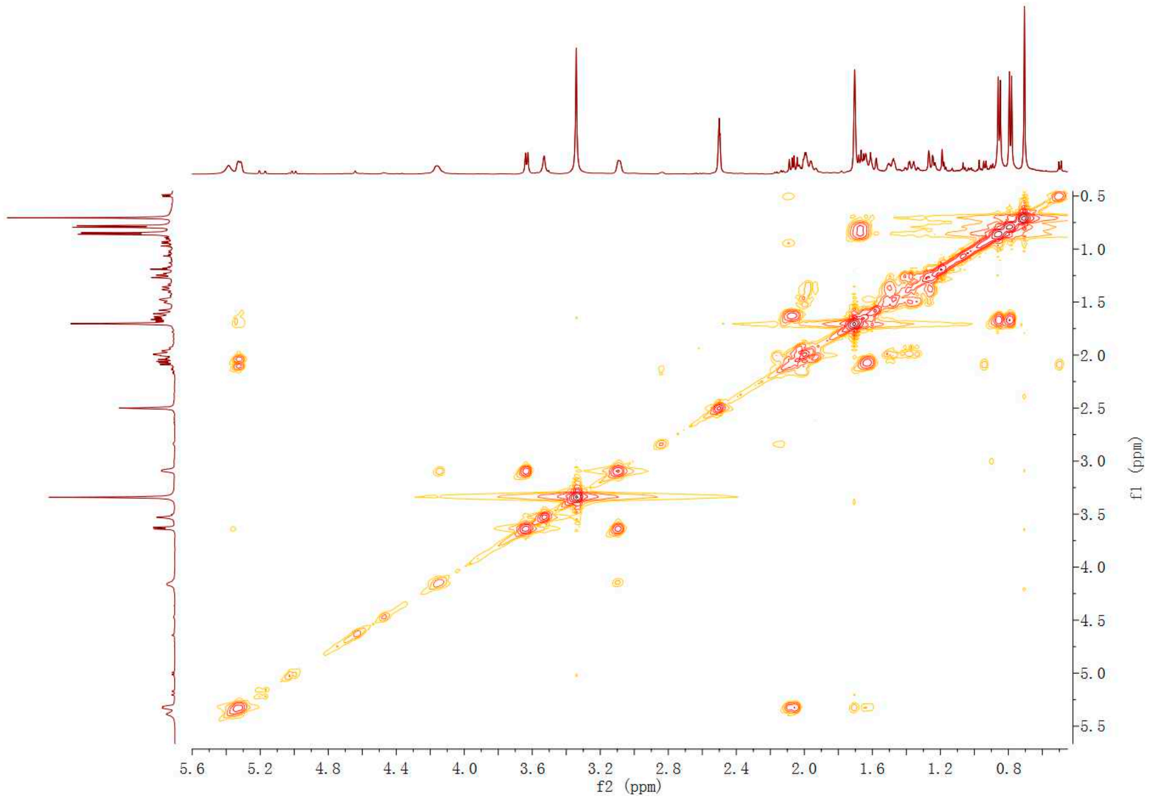


Figure S4. HSQC spectrum of compound **1**;

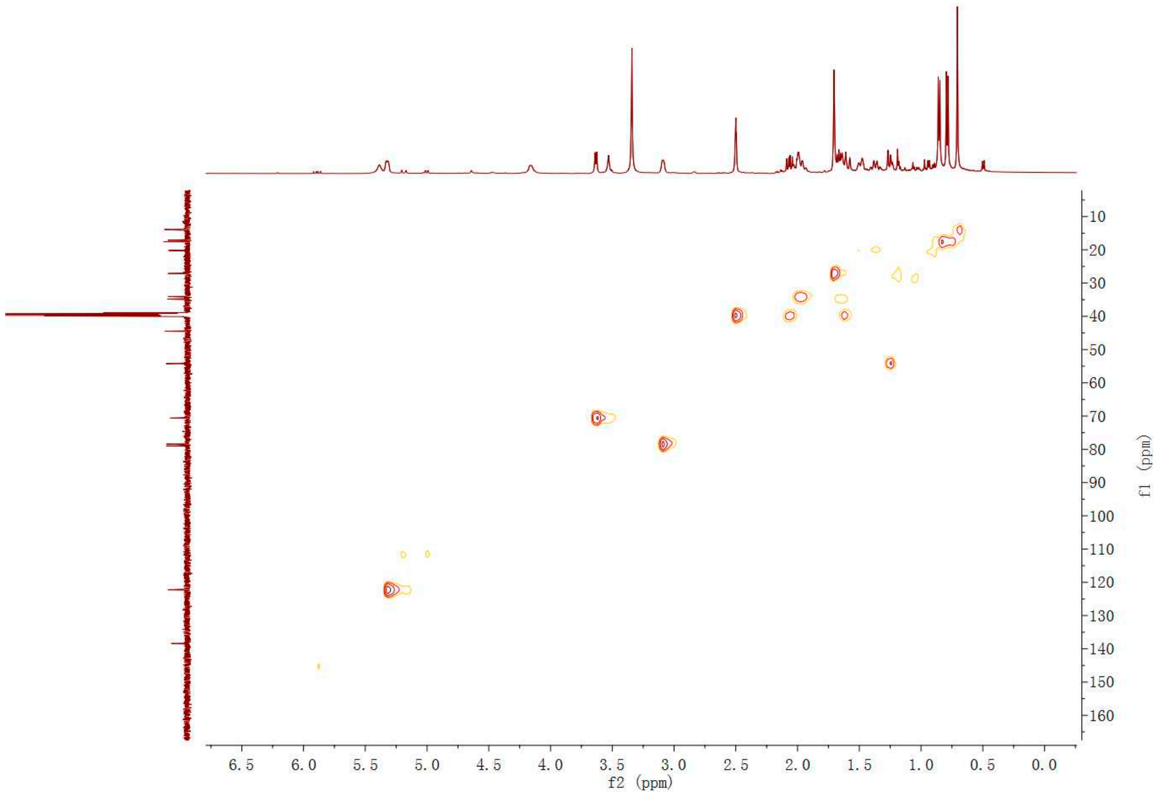

Figure S5. HMBC spectrum of compound **1**;

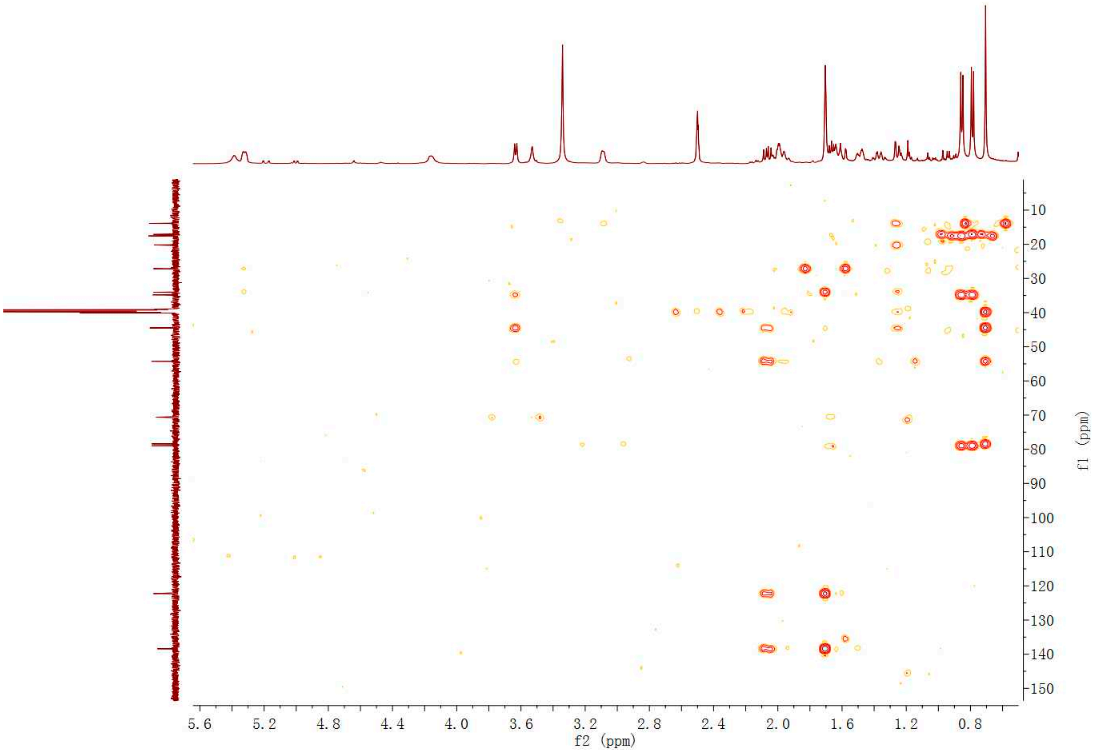


Figure S6. NOESY spectrum of compound **1**;

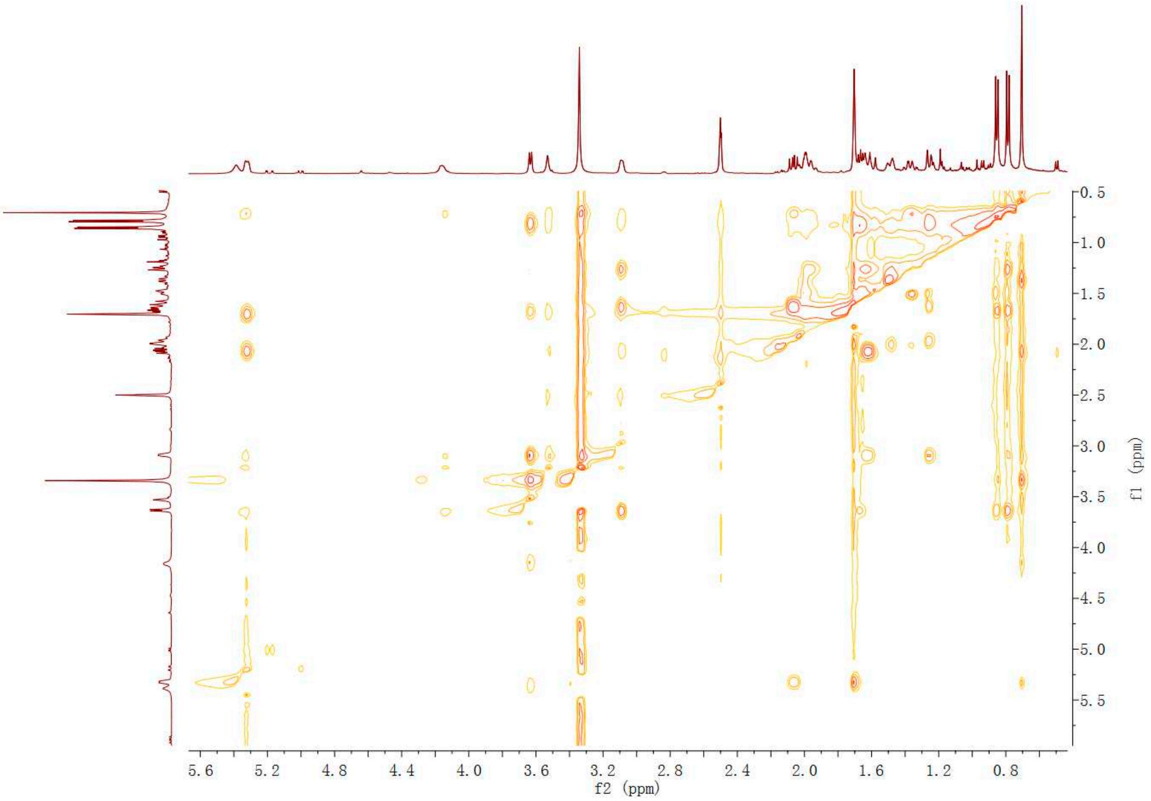

Figure S7. HRESIMS spectrum of compound **1**;

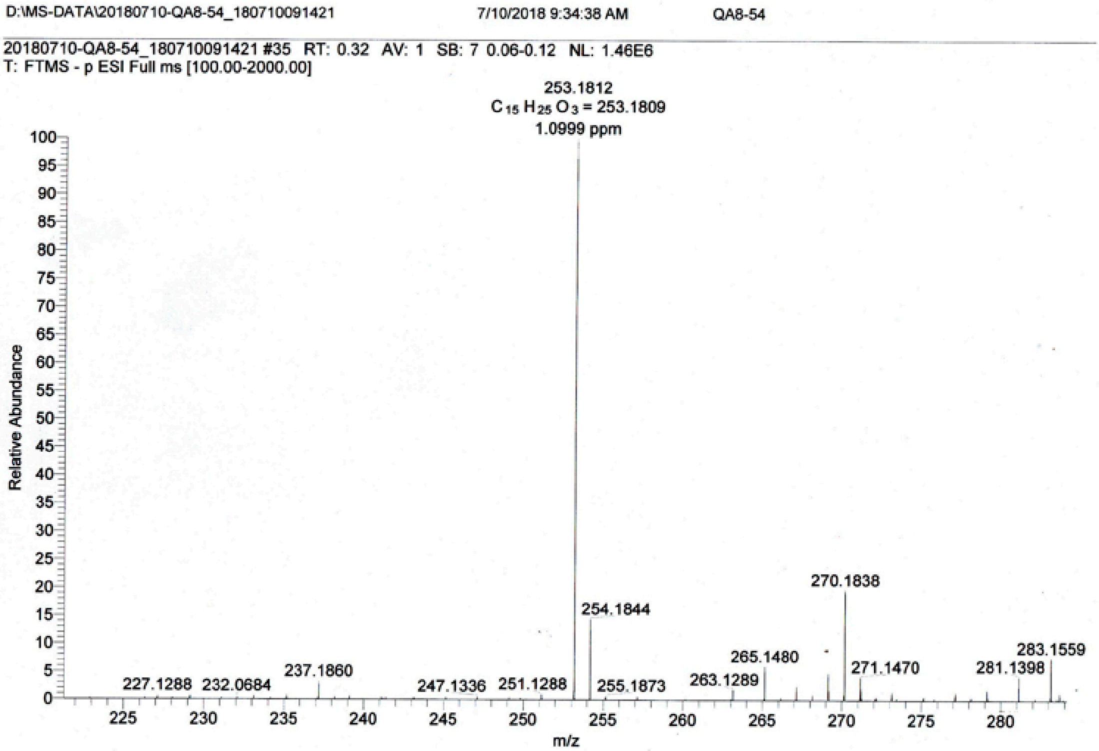


Figure S8. $^{1}$H NMR (500 MHz, DMSO-$d_6$) spectrum of compound **2**;

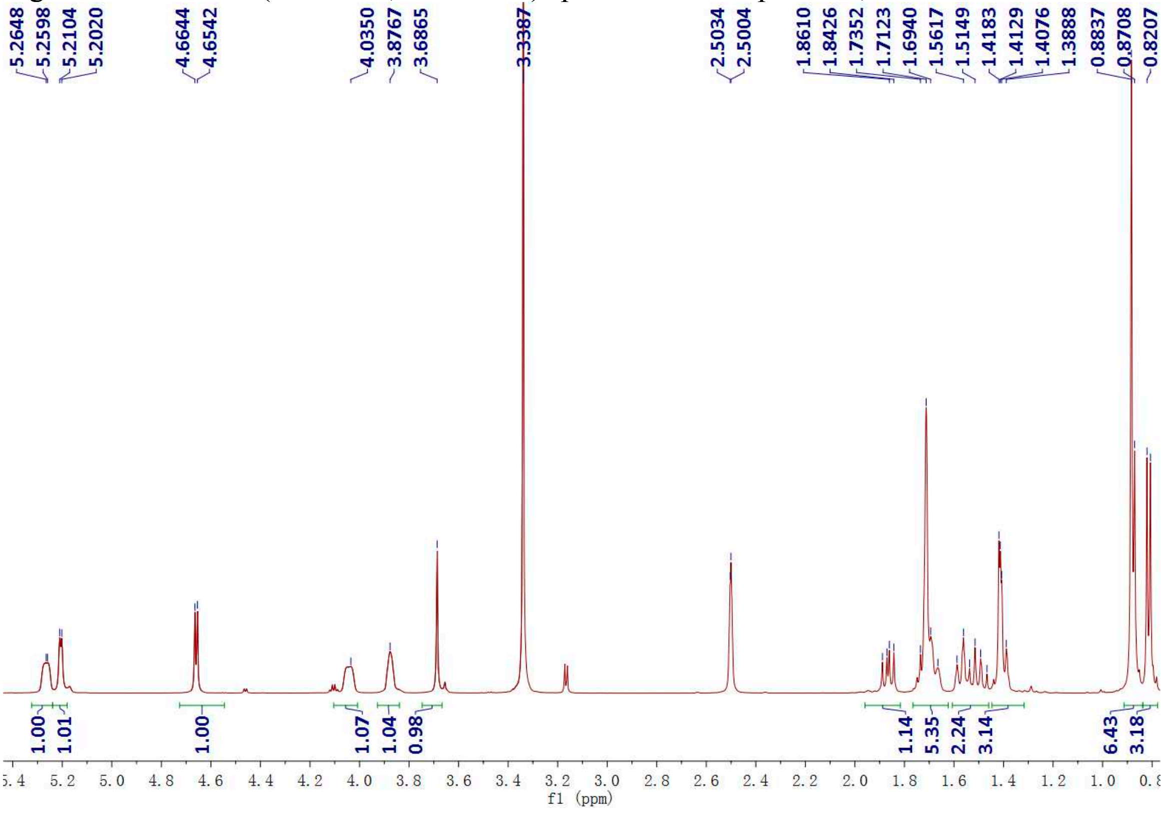

Figure S9. $^{13}C$ NMR (125 MHz, DMSO-$d_6$) and DEPT spectra of compound **2**;

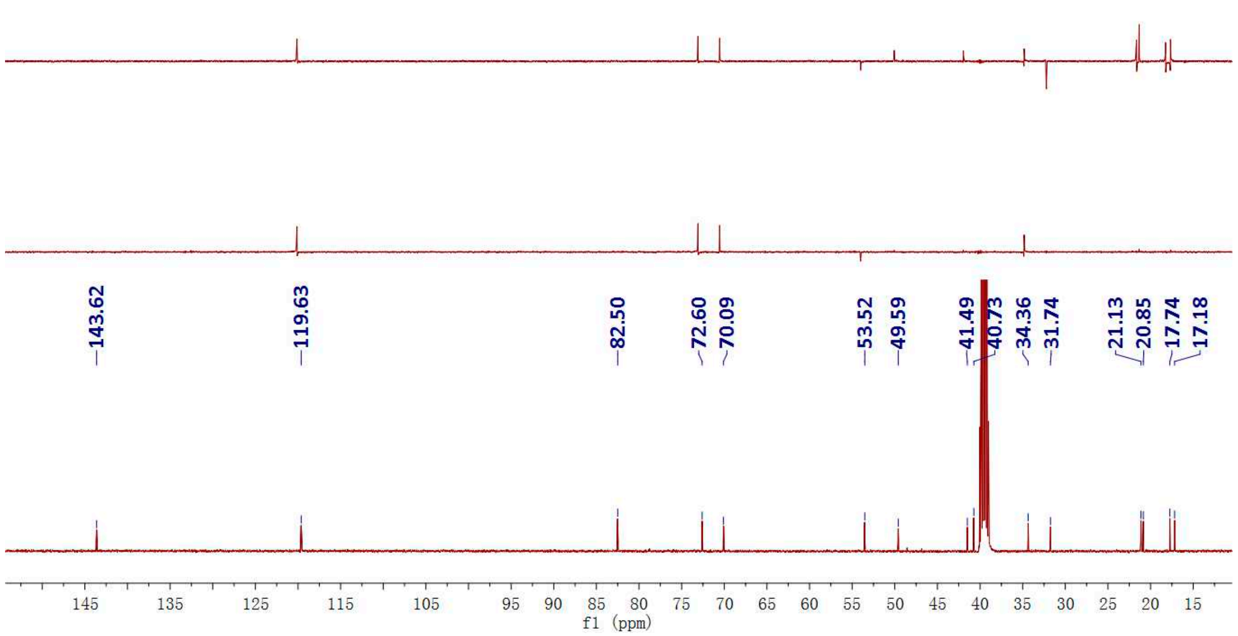


Figure S10. $^1H$ -$^1H$ COSY spectrum of compound **2**;

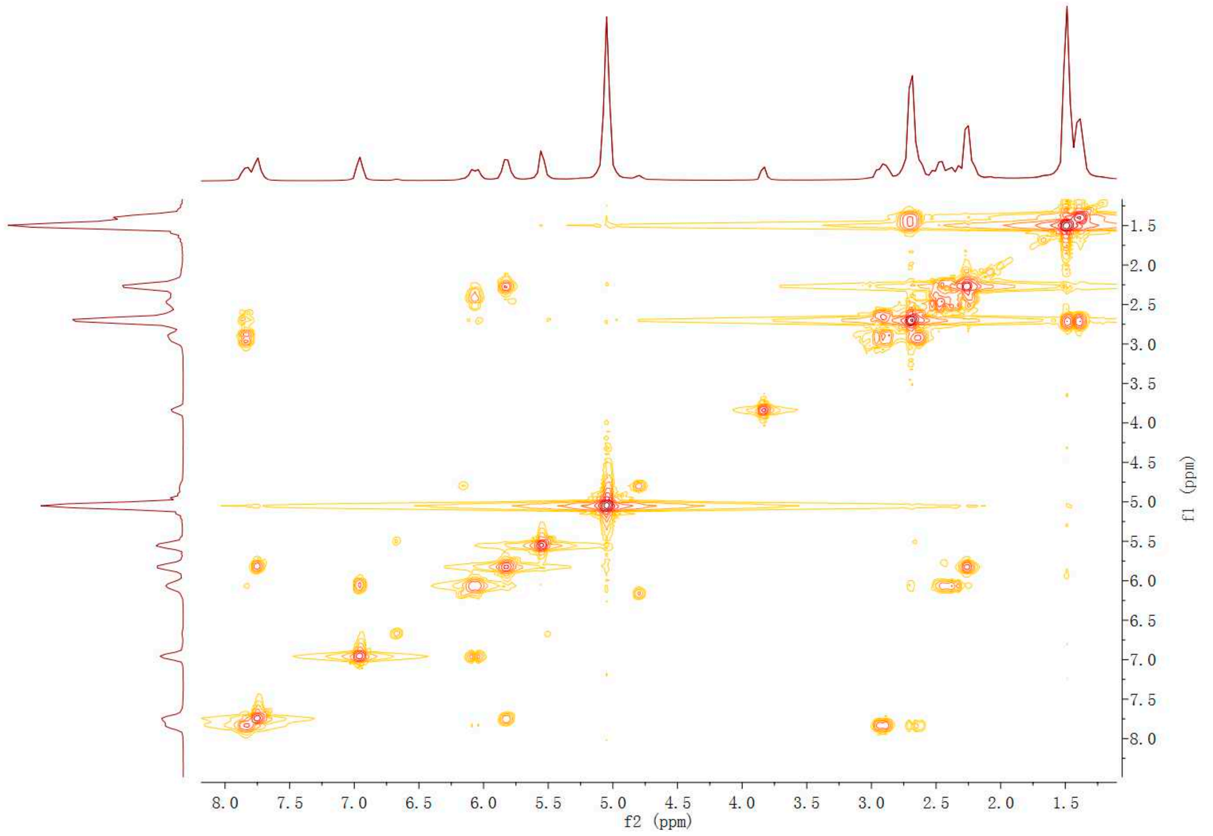

Figure S11. HSQC spectrum of compound **2**;

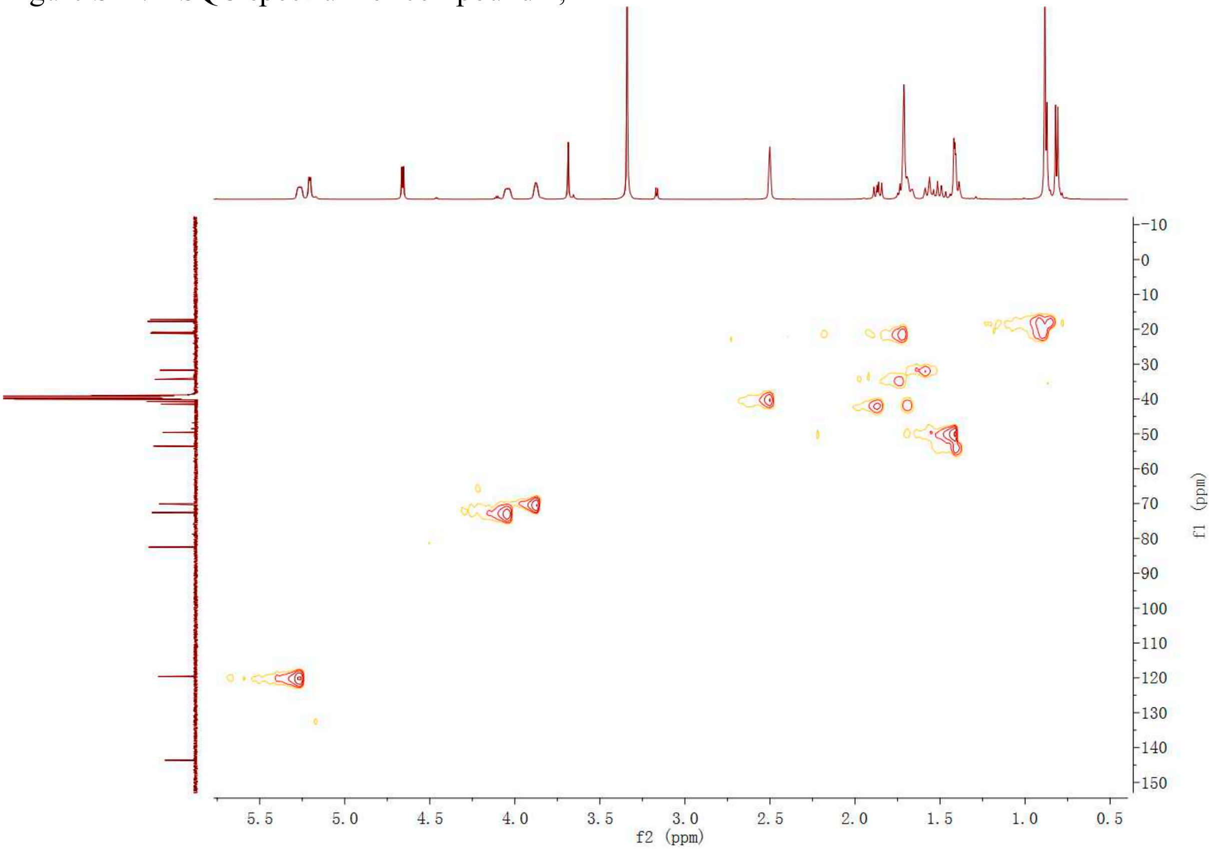


Figure S12. HMBC spectrum of compound **2**;

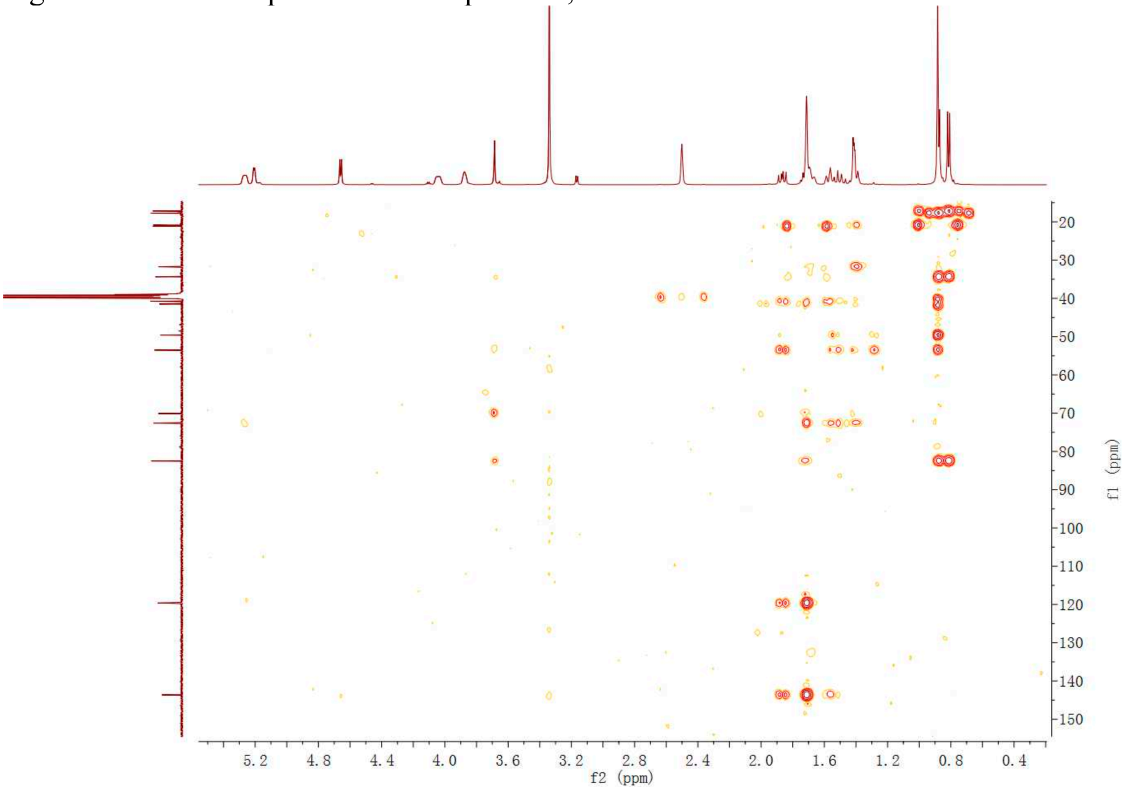

Figure S13. NOESY spectrum of compound **2**;

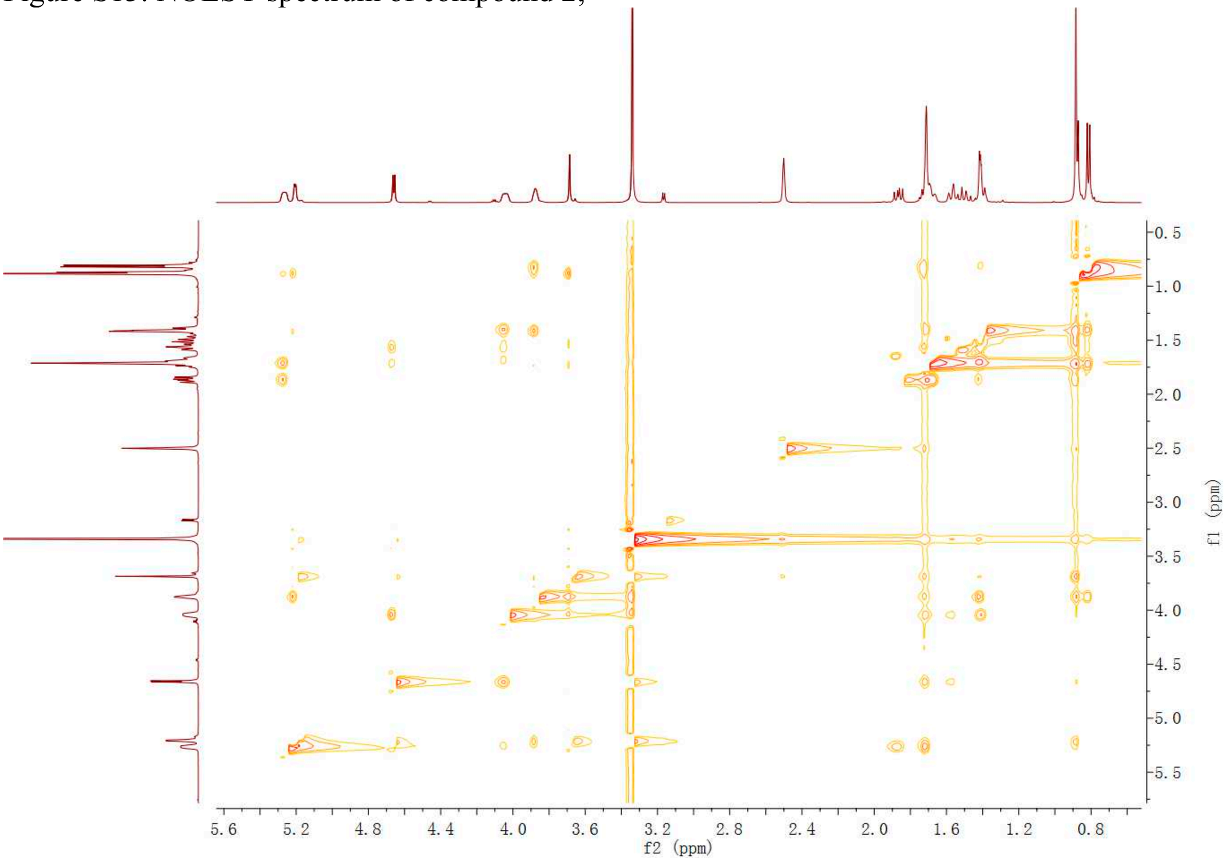


Figure S14. HRESIMS spectrum of compound **2**;

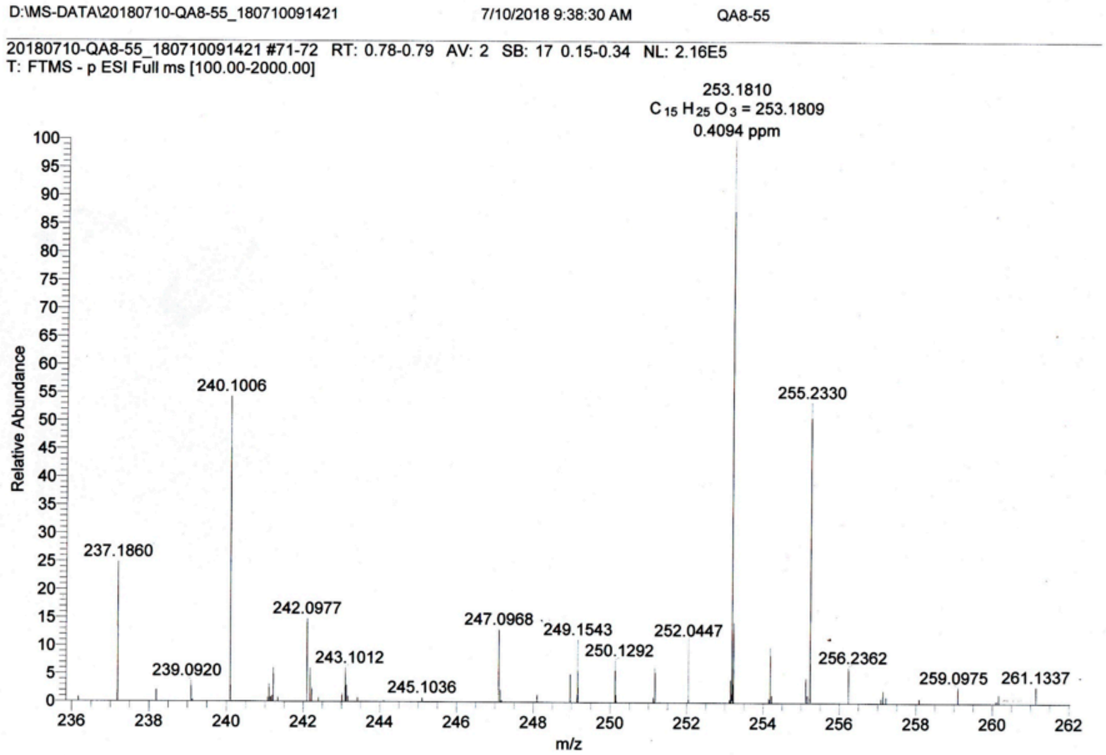

Figure S15. $^{1}$H NMR (500 MHz, DMSO-$d_6$) spectrum of compound **3**;

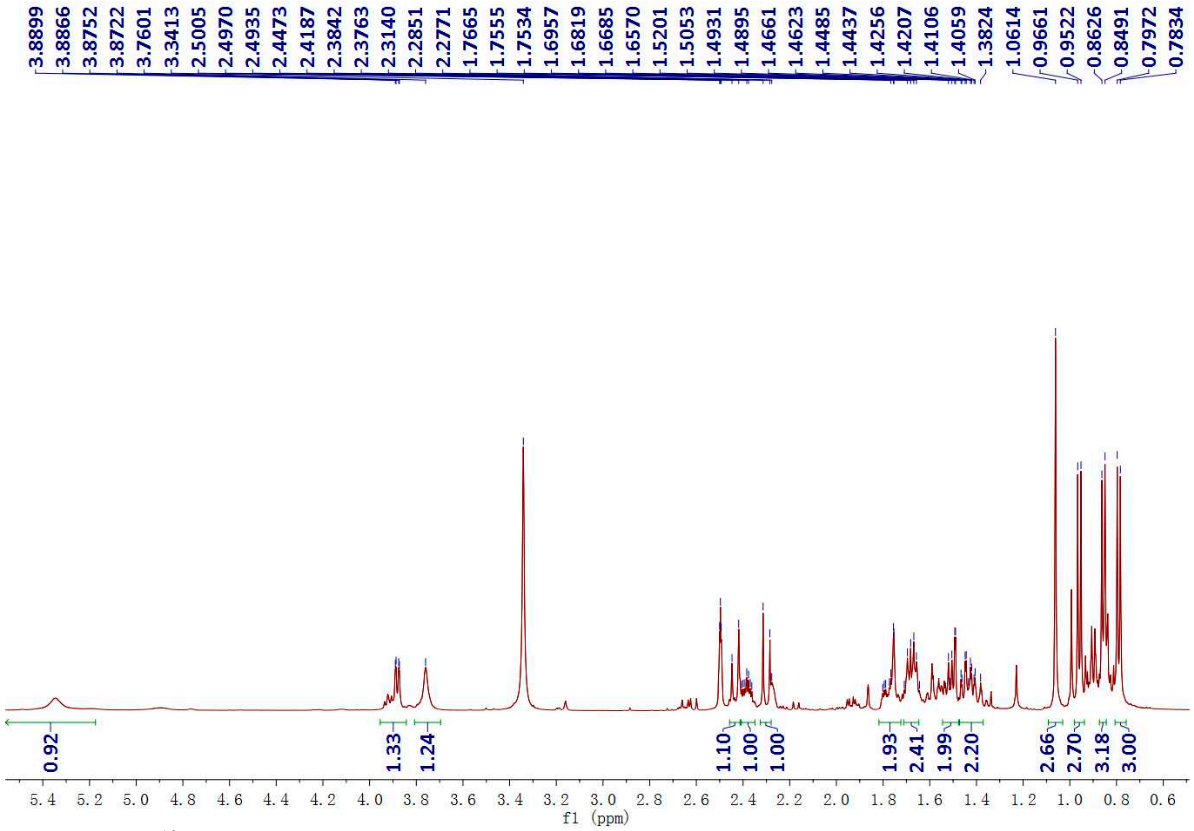


Figure S16. $^{13}$C NMR (125 MHz, DMSO-$d_6$) and DEPT spectra of compound **3**;

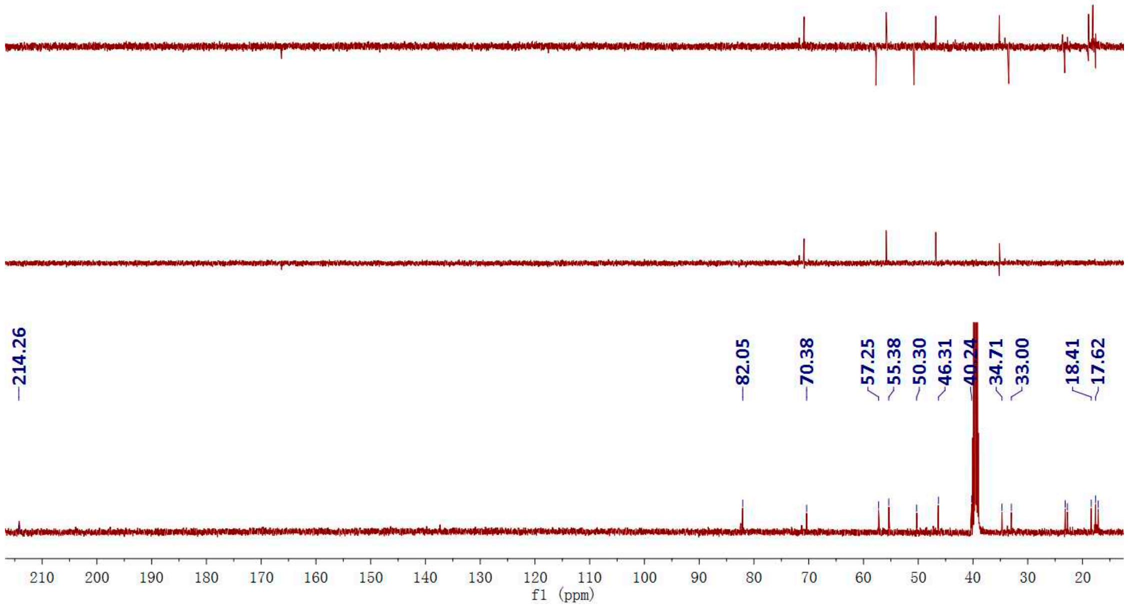

Figure S17. $^1$H -$^1$H COSY spectrum of compound **3**;

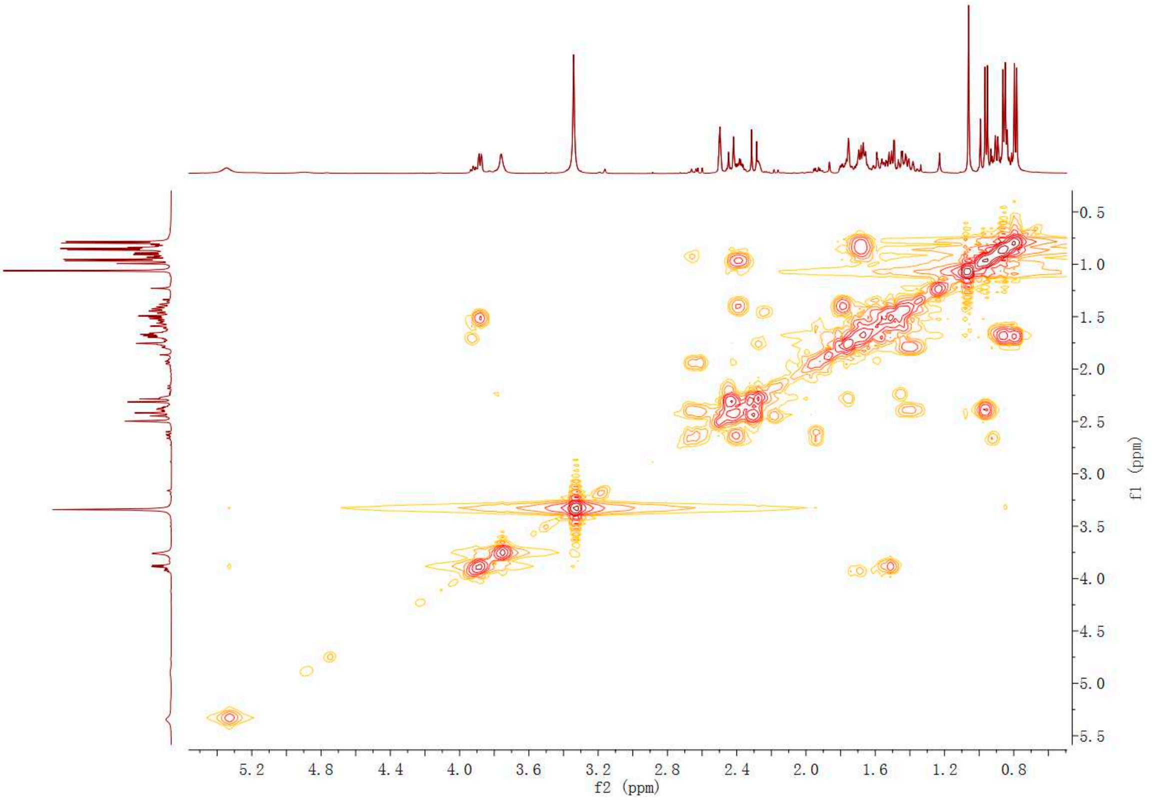


Figure S18. HSQC spectrum of compound **3**;

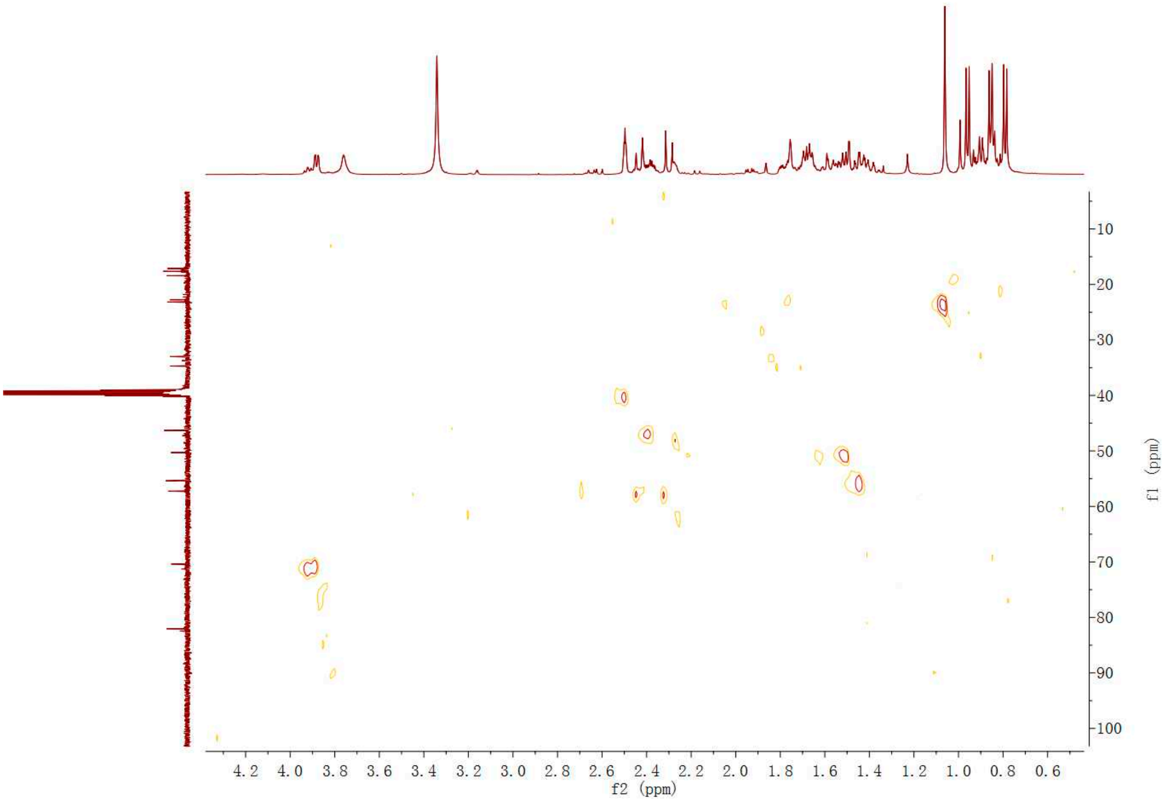

Figure S19. HMBC spectrum of compound **3**;

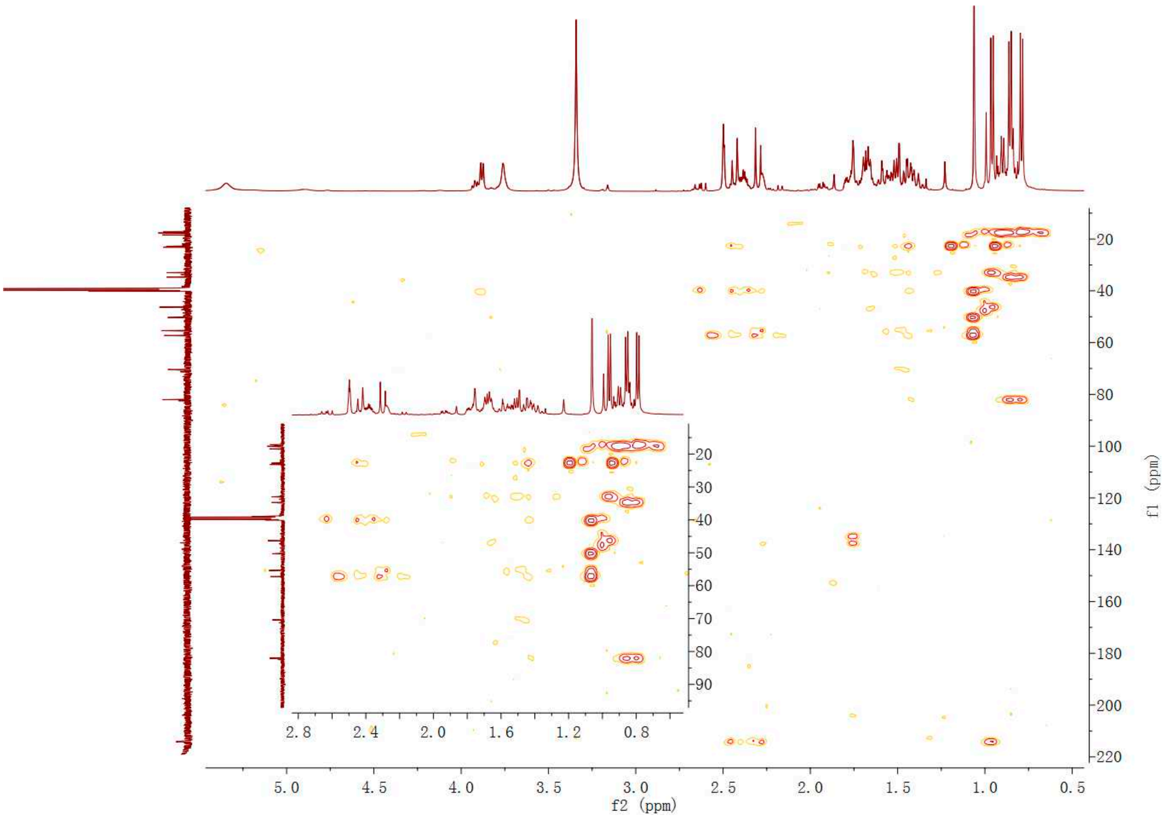


Figure S20. NOESY spectrum of compound **3**;

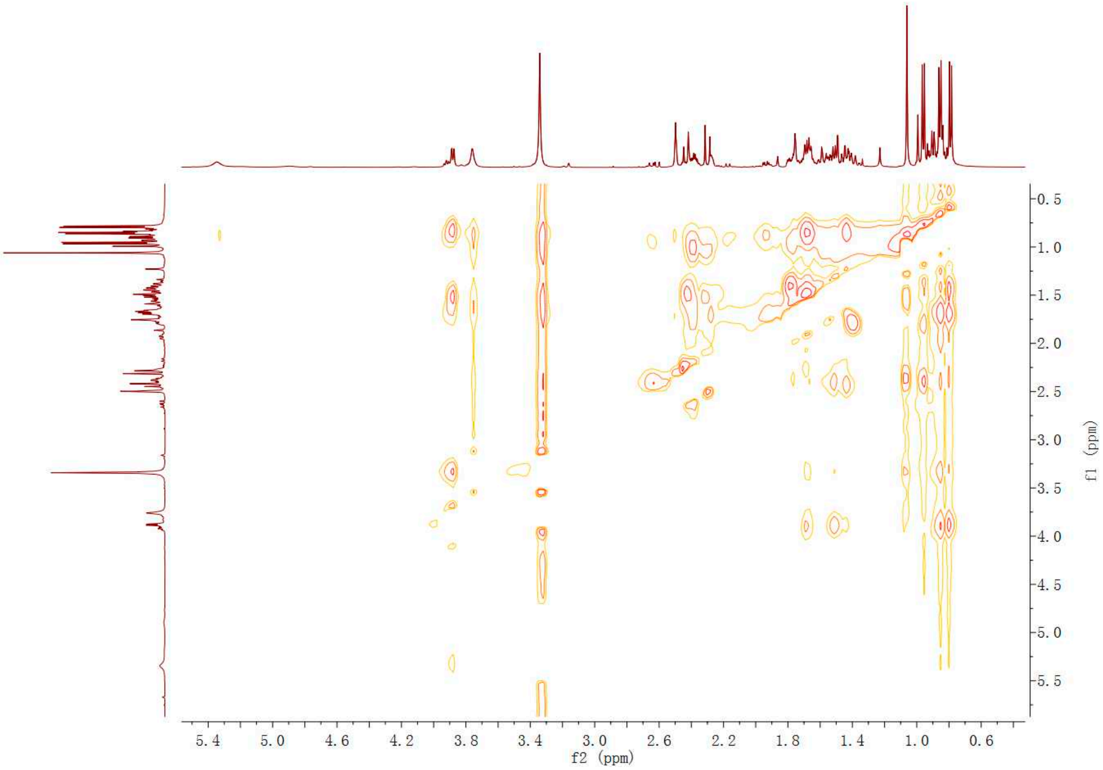

Figure S21. HRESIMS spectrum of compound **3**;

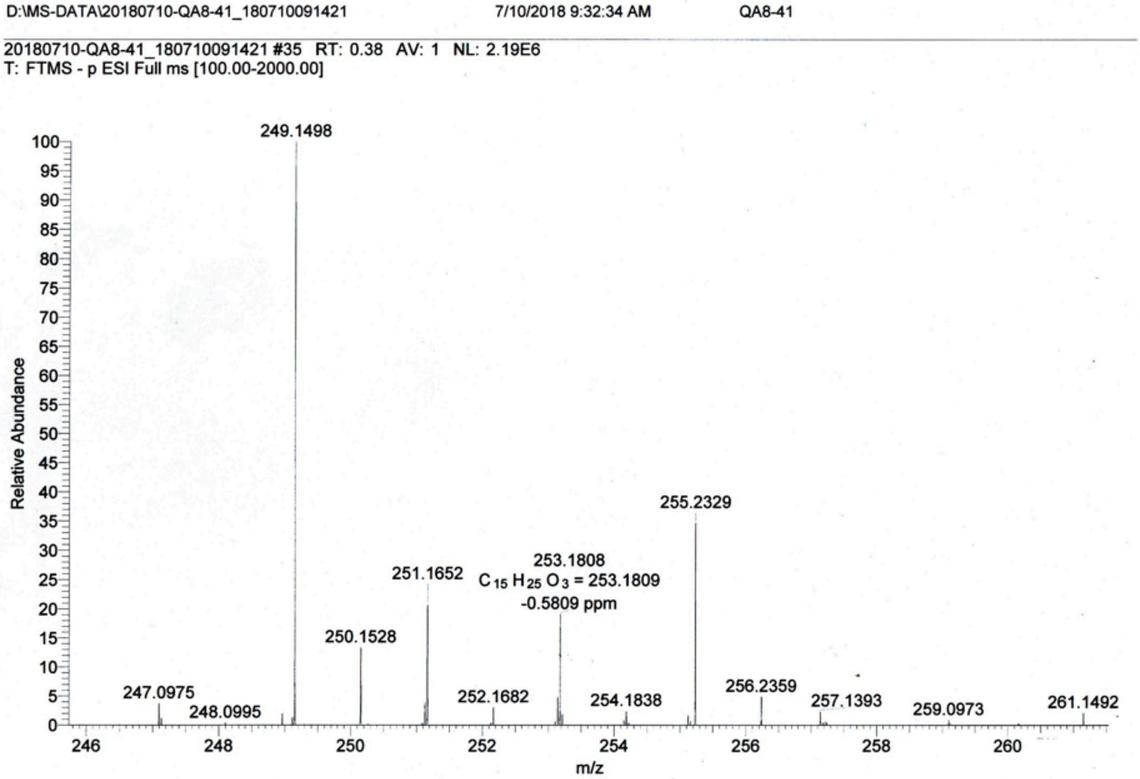


Figure S22. $^{1}$H NMR (500 MHz, DMSO-$d_6$) spectrum of compound **4**;

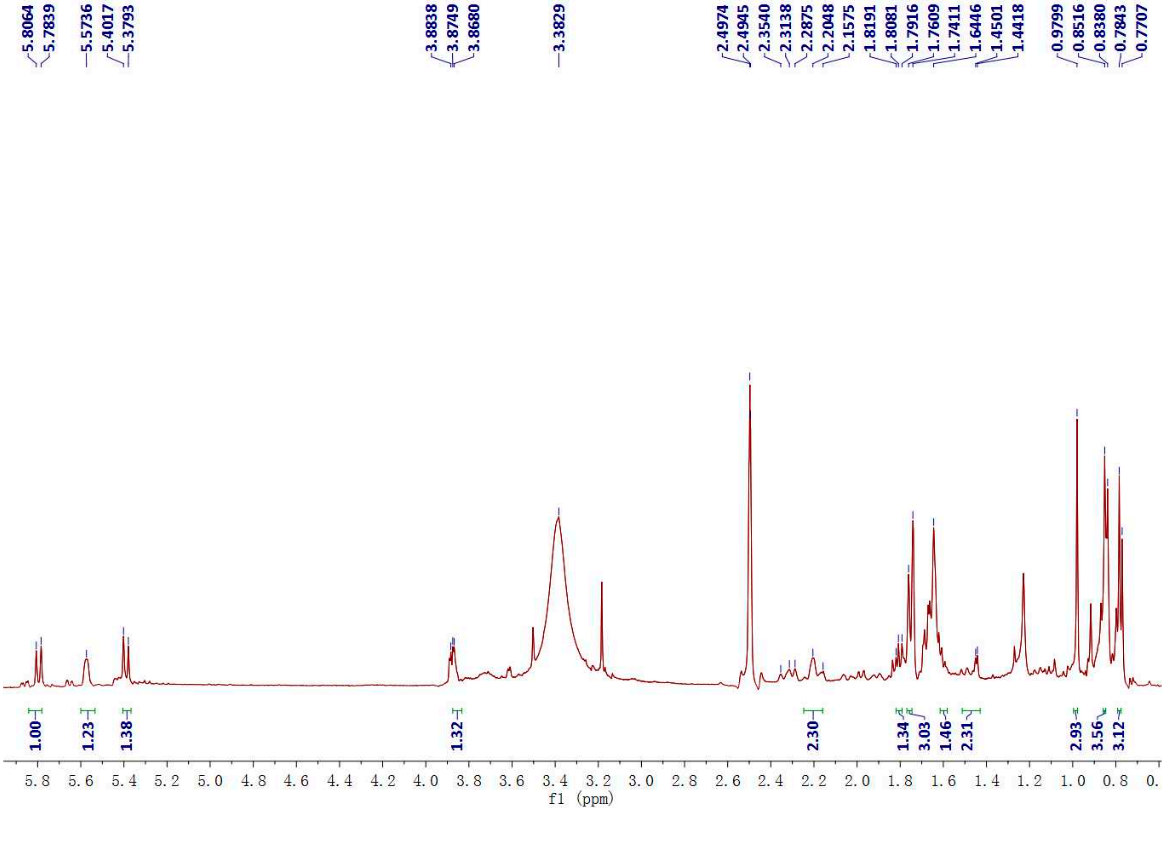

Figure S23. $^{13}C$ NMR (125 MHz, DMSO-$d_6$) and DEPT spectra of compound **4**;

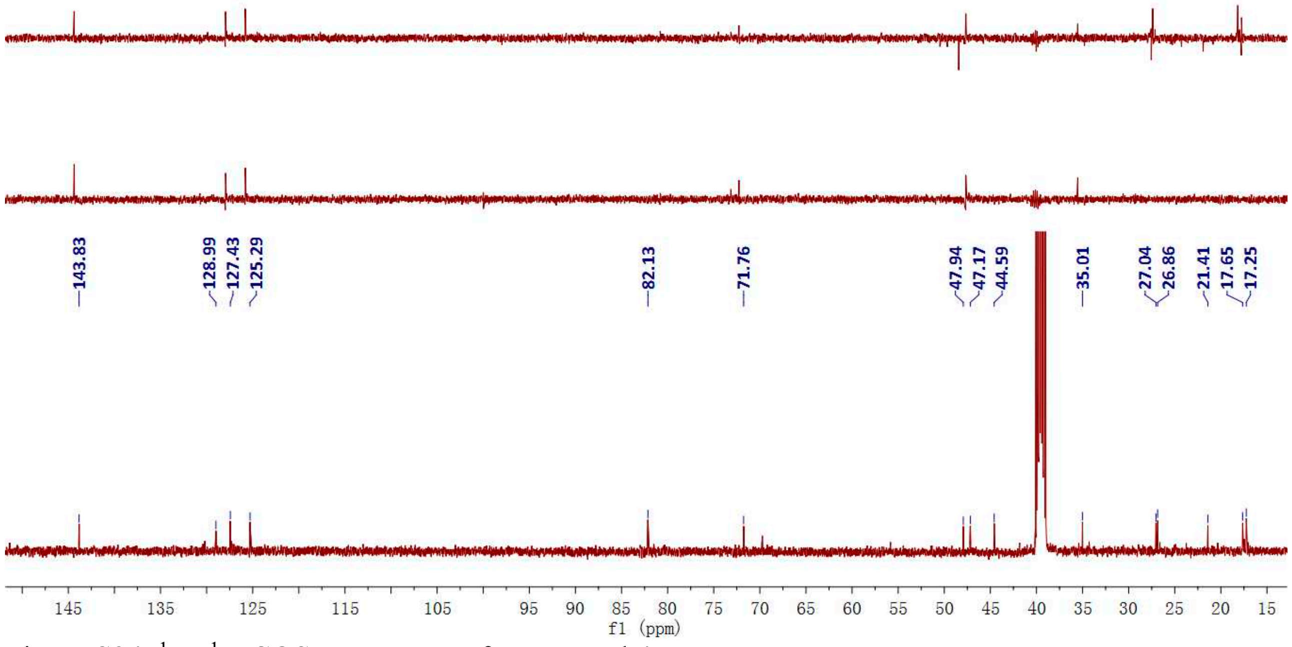


Figure S24. $^{1}H$ -$^{1}H$ COSY spectrum of compound **4**;

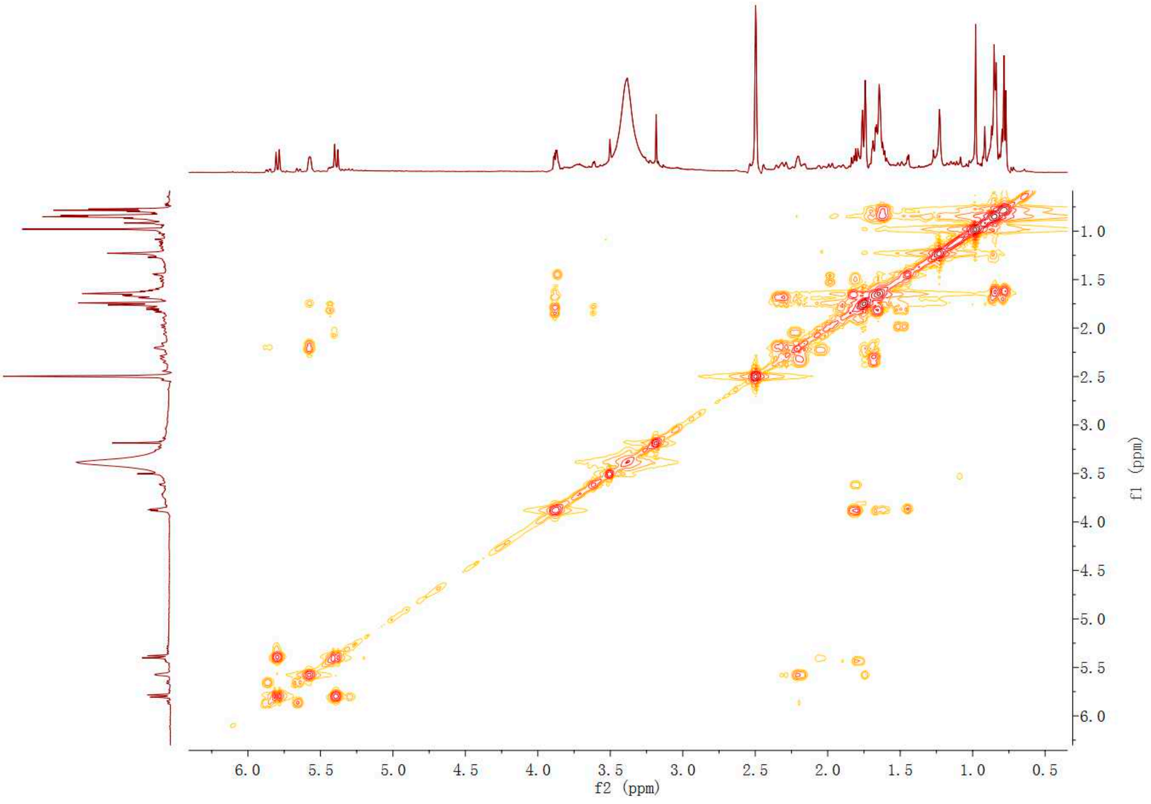

Figure S25. HSQC spectrum of compound **4**;

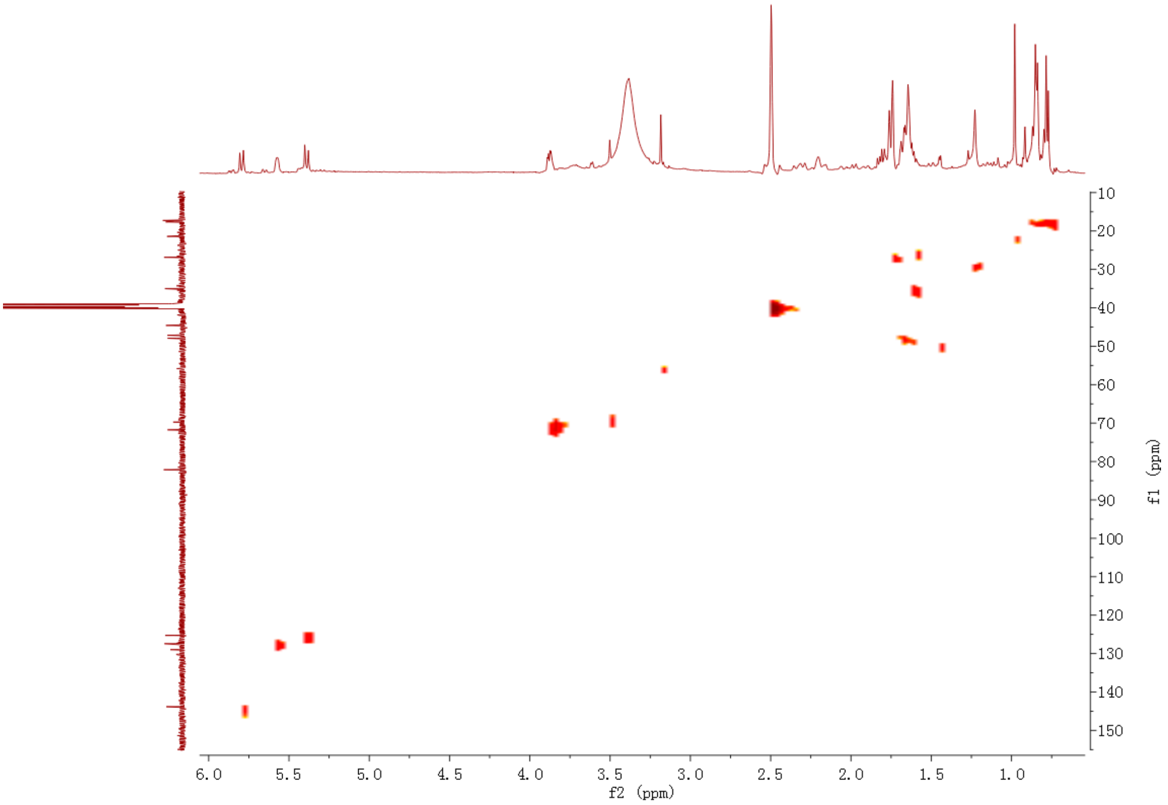


Figure S26. HMBC spectrum of compound **4**;

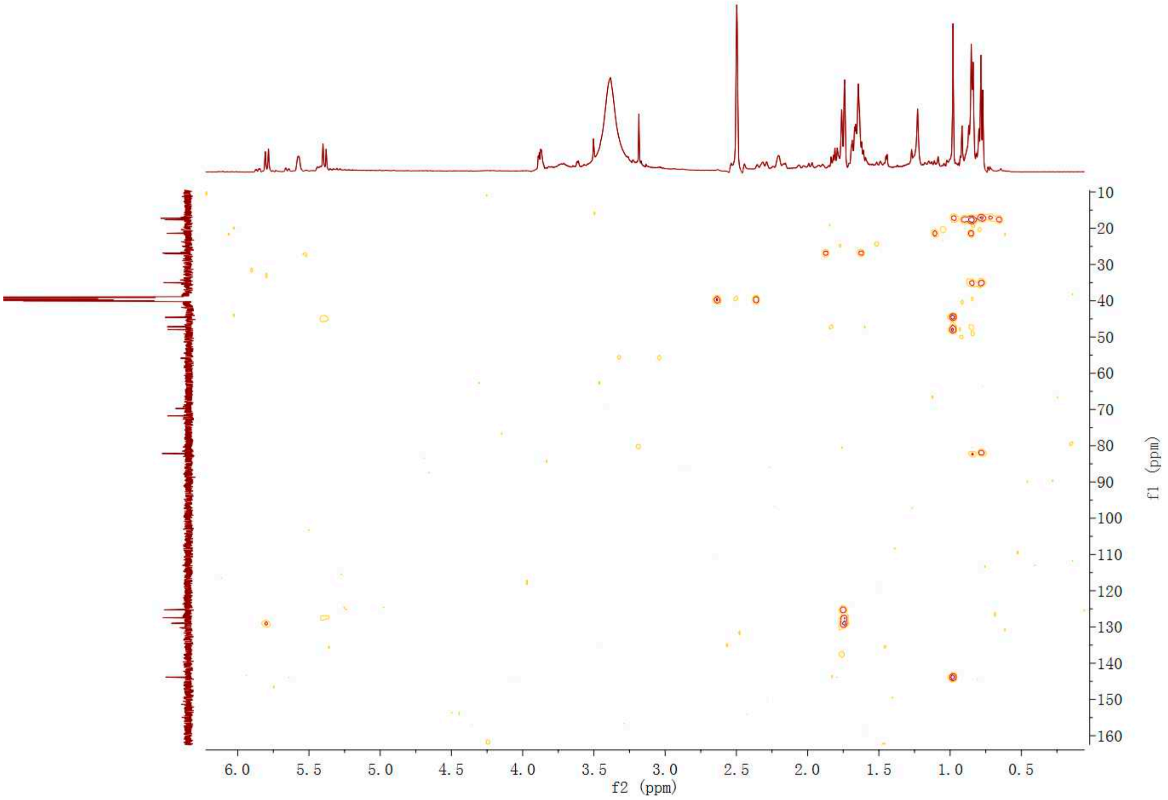

Figure S27. NOESY spectrum of compound **4**;

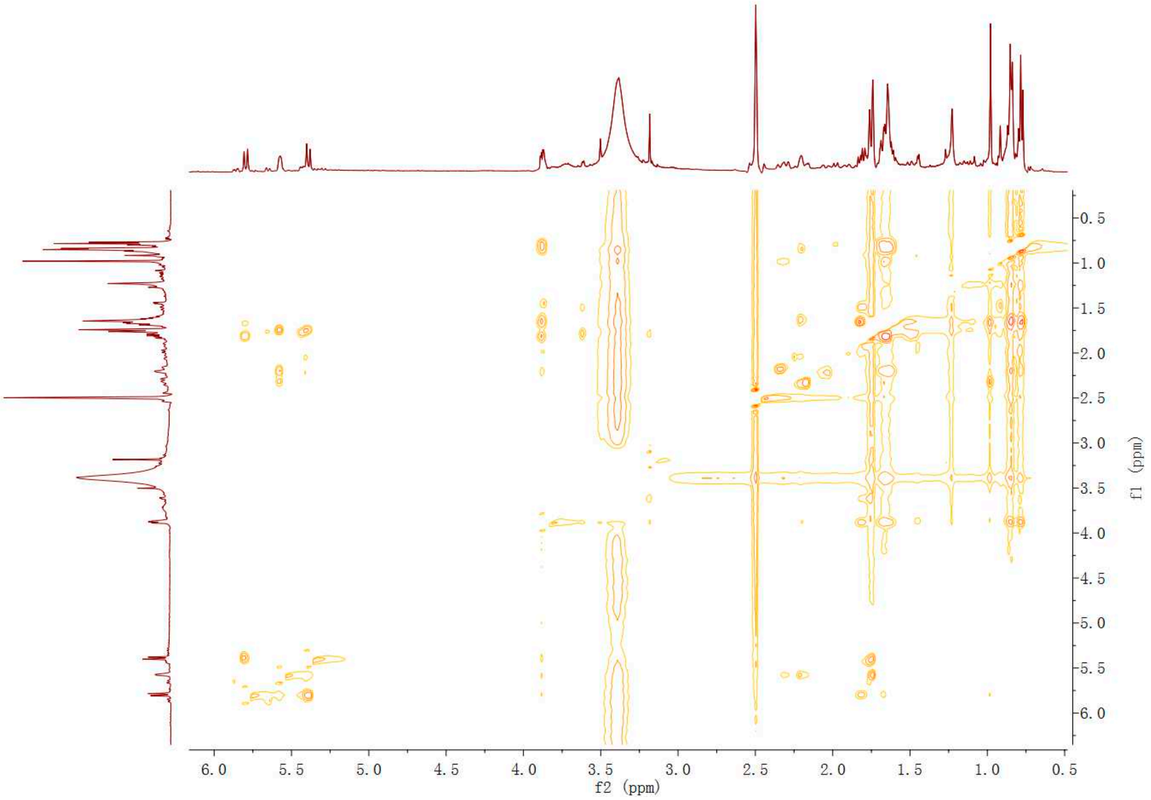


Figure S28. HRESIMS spectrum of compound **4**;

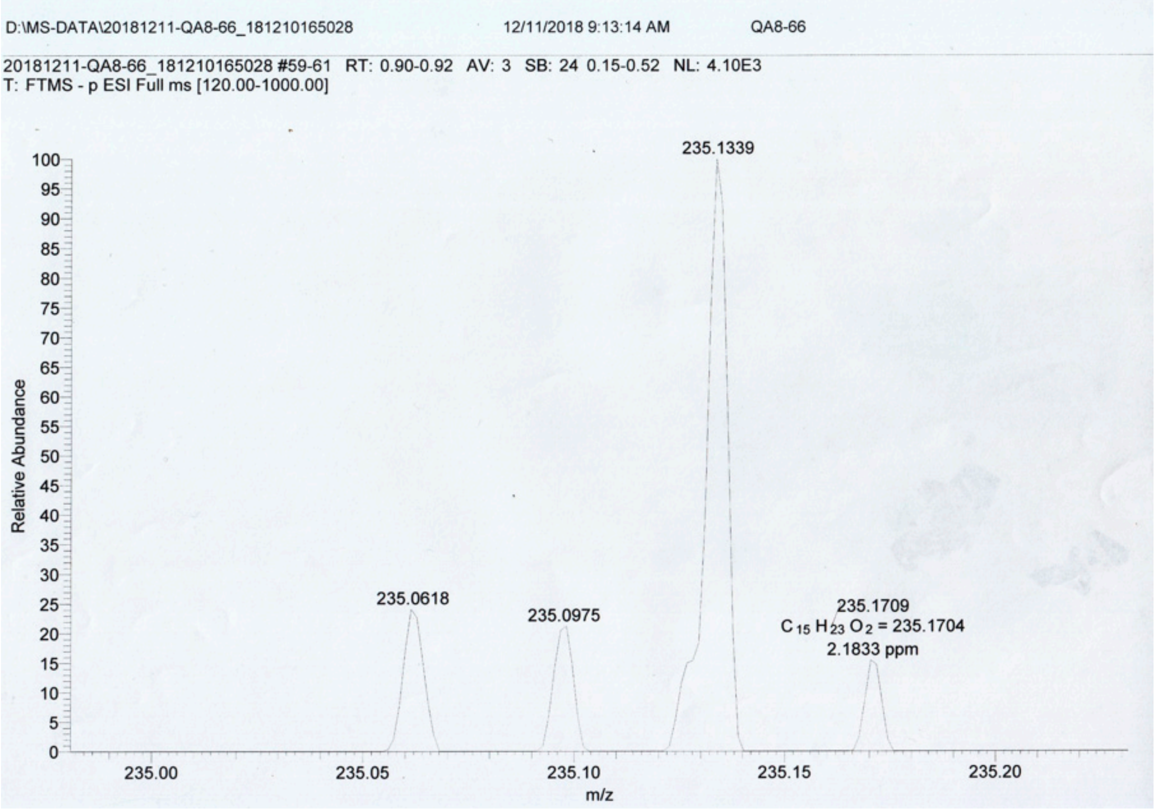

Figure S29.$^{1}$H NMR (500 MHz, DMSO-$d_6$) spectrum of compound **5**;

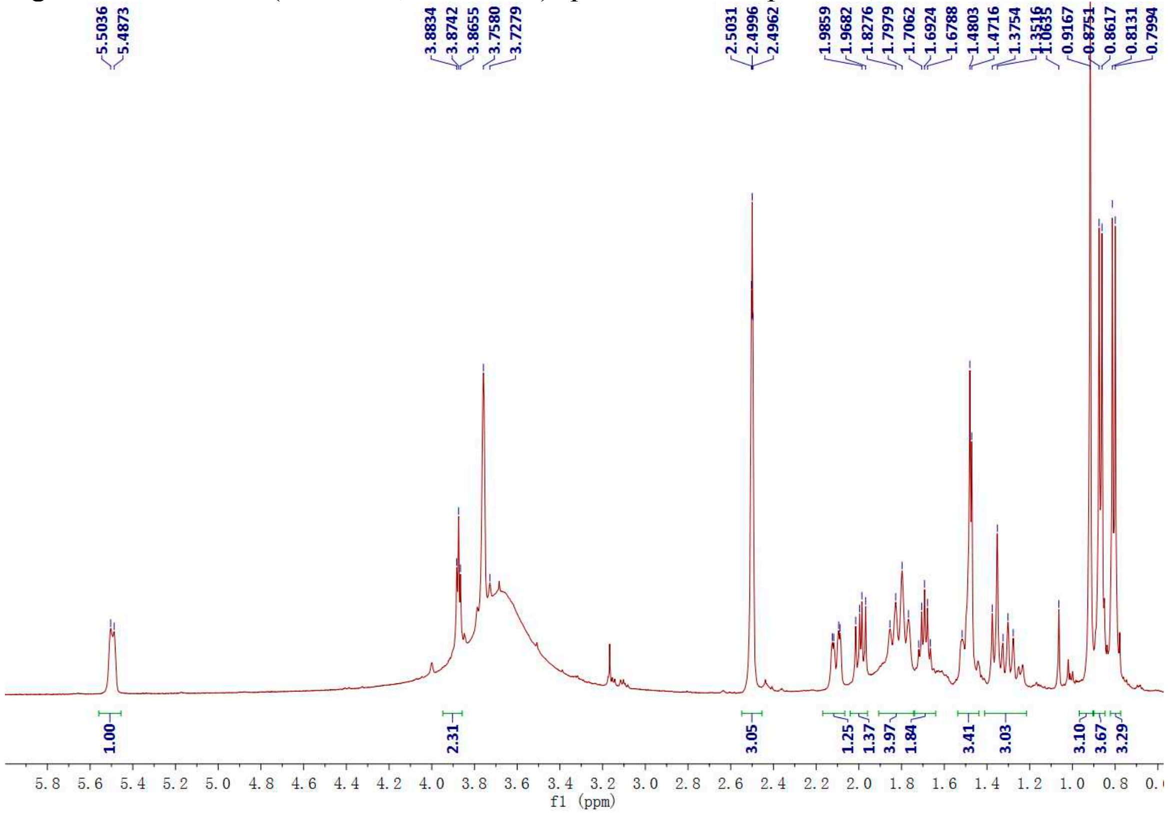


Figure S30. $^{13}$C NMR (125 MHz, DMSO-$d_6$) and DEPT spectra of compound **5**;

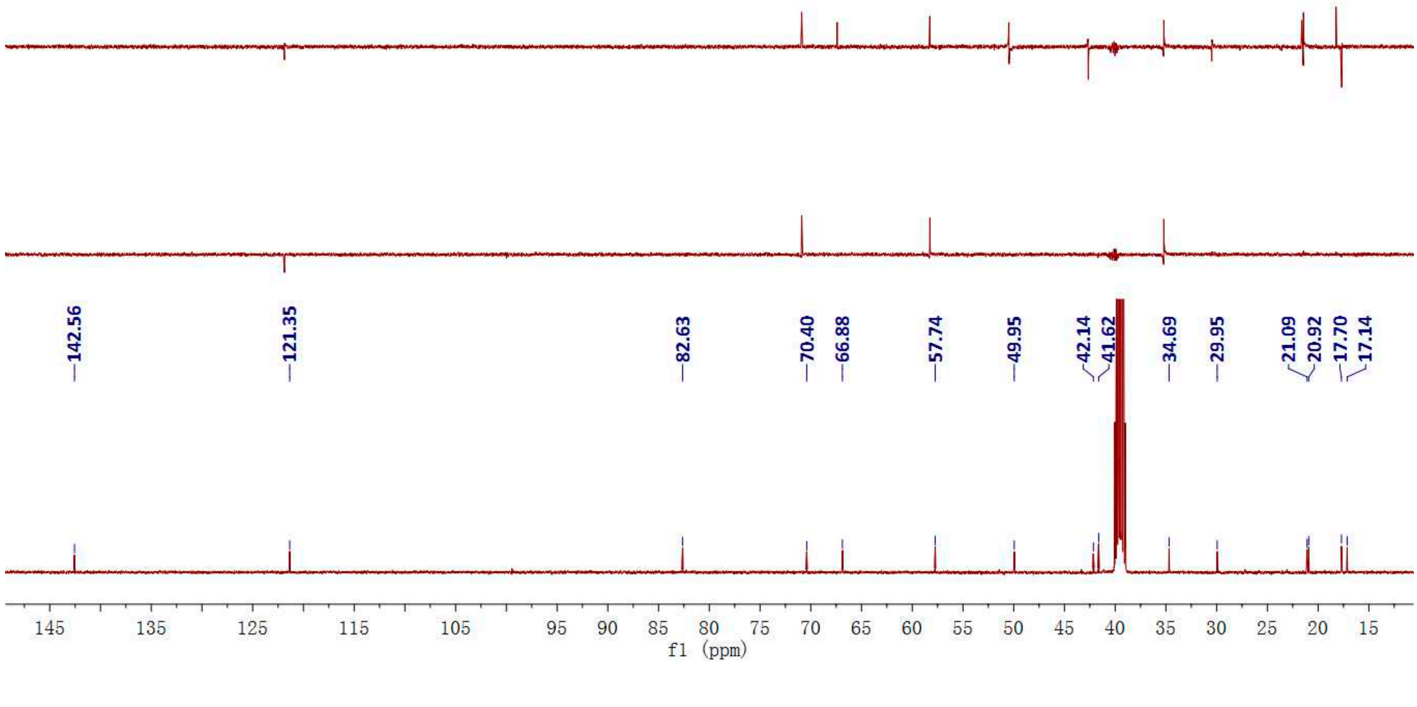

Figure S31. COSY spectrum of compound **5**;

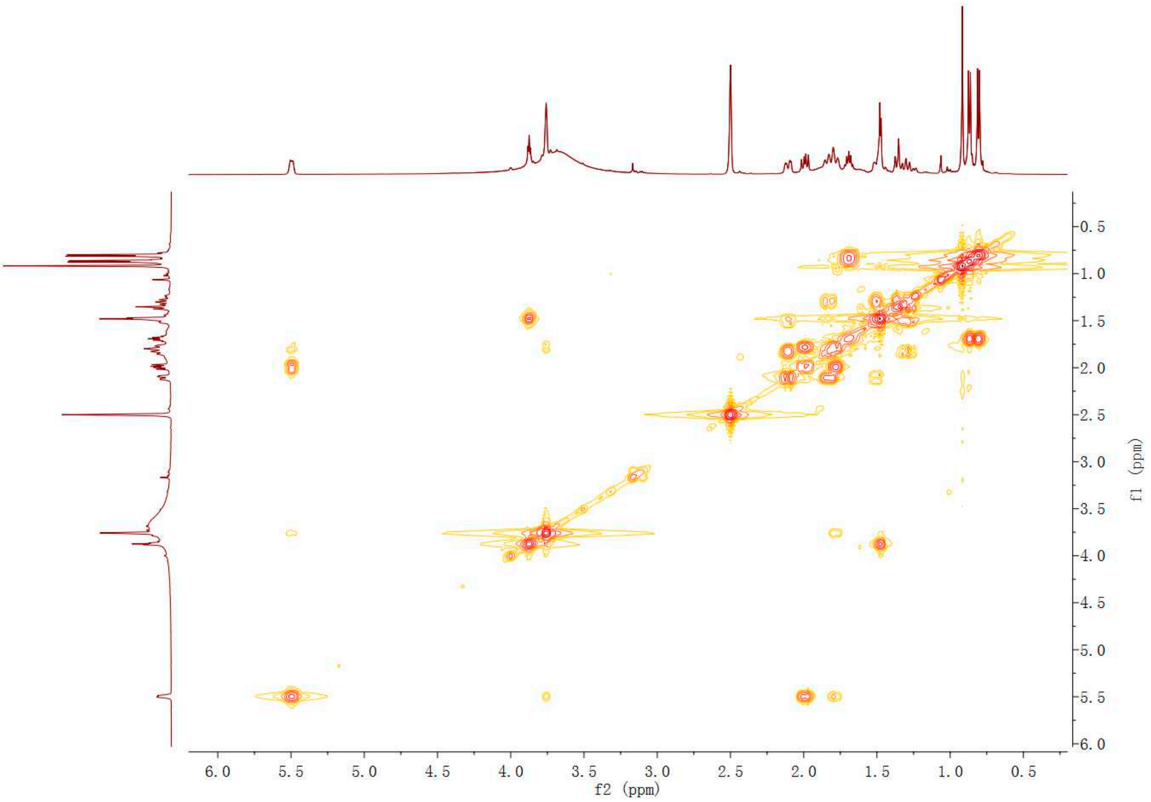


Figure S32. HSQC spectrum of compound **5**;

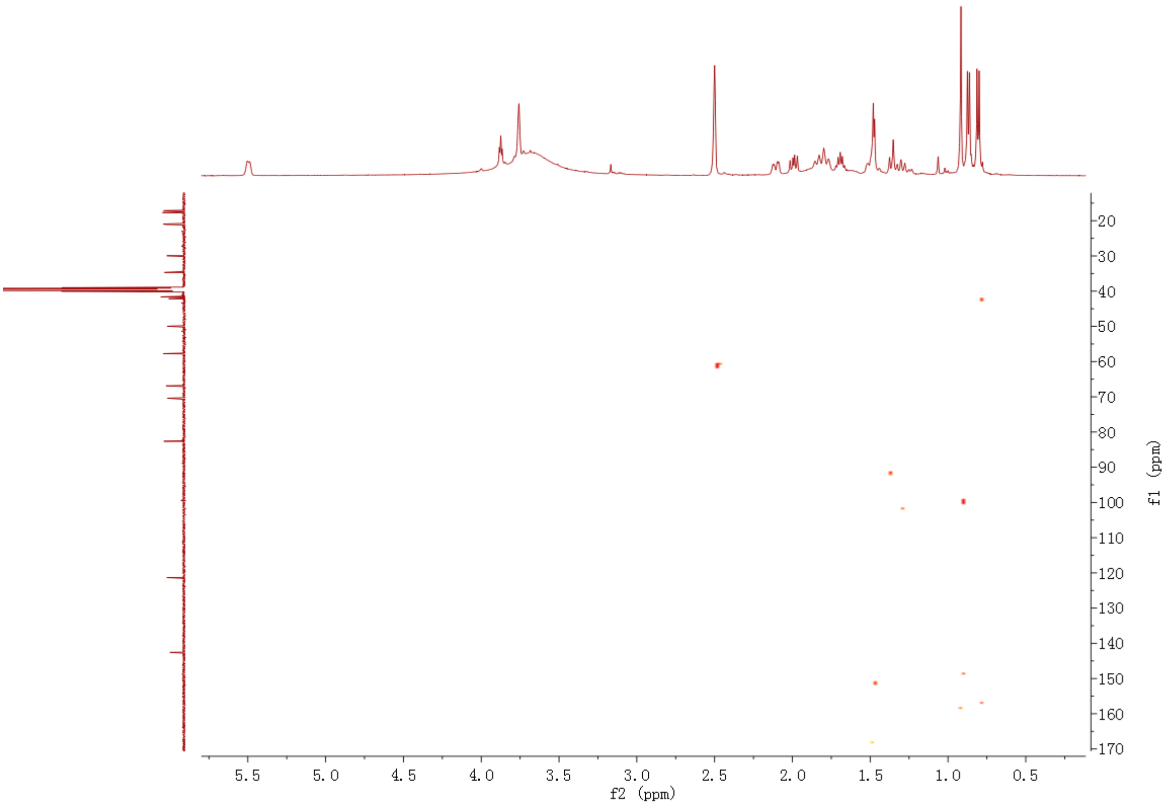

Figure S33. HMBC spectrum of compound **5**;

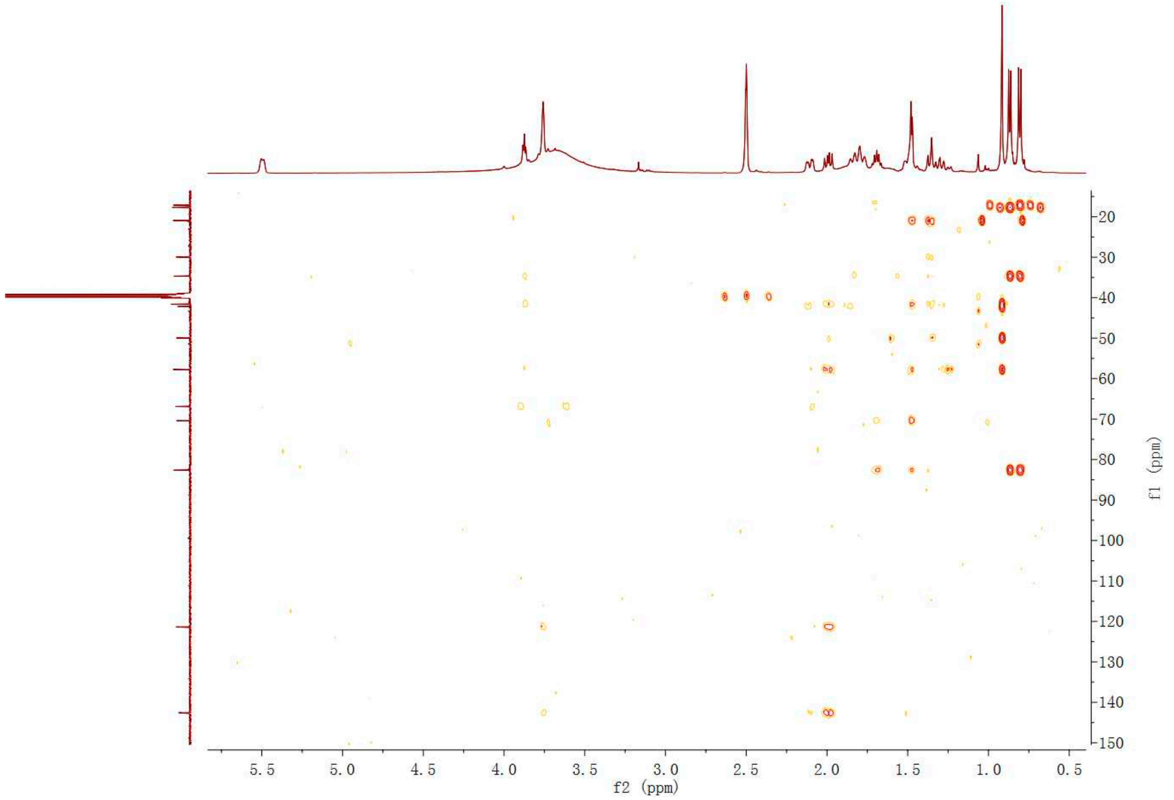


Figure S34. NOESY spectrum of compound **5**;

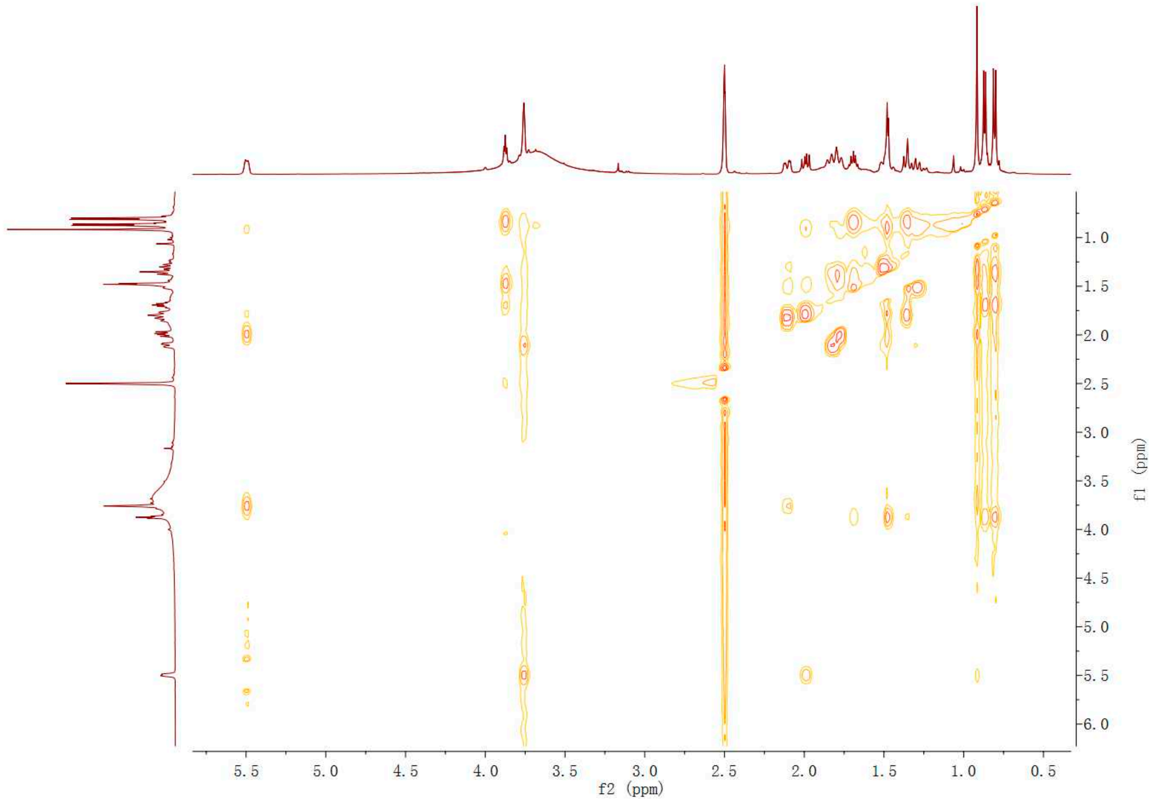

Figure S35. HRESIMS spectrum of compound **5**;

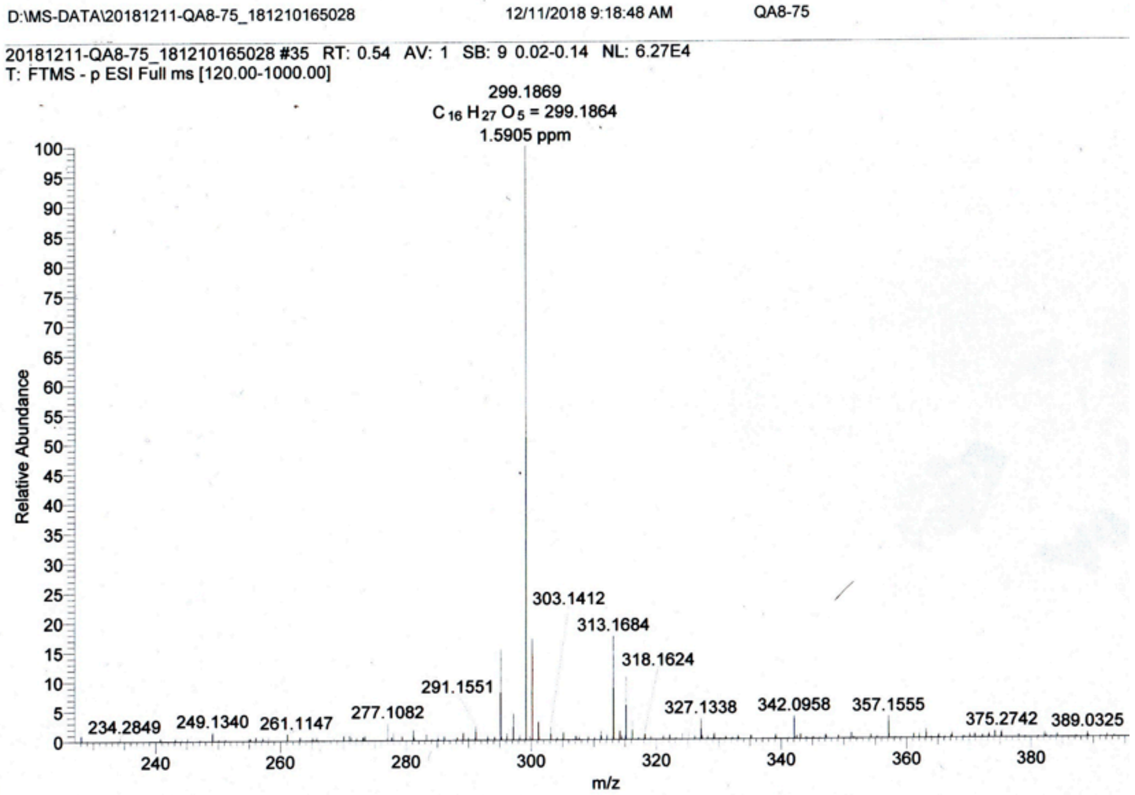


Figure S36. Crystal packing of compound **1** at 293(2) K;

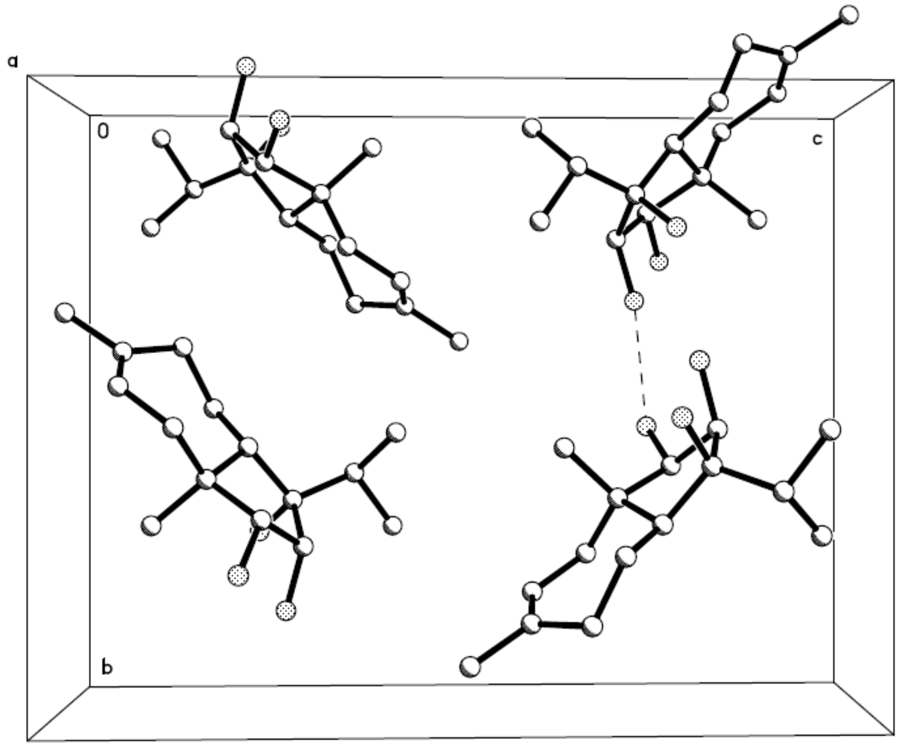

Figure S37. Crystal packing of compound **9** at 293(2) K;

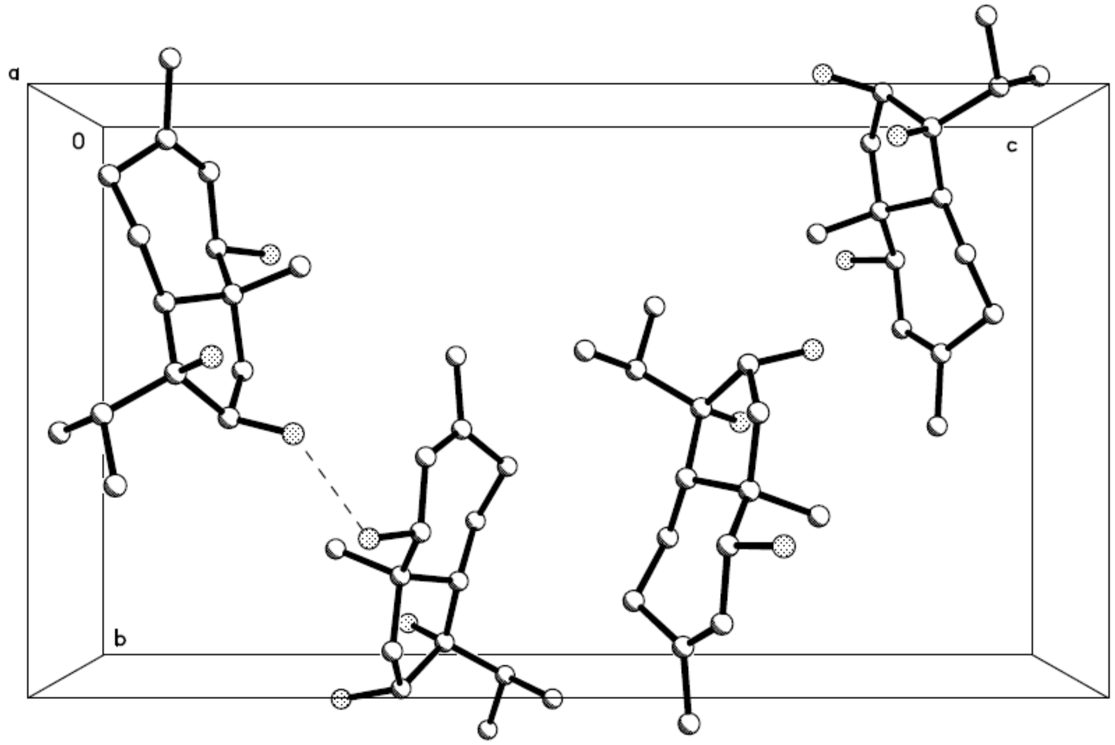


Figure S38. Crystal packing of compound **10** at 293(2) K.

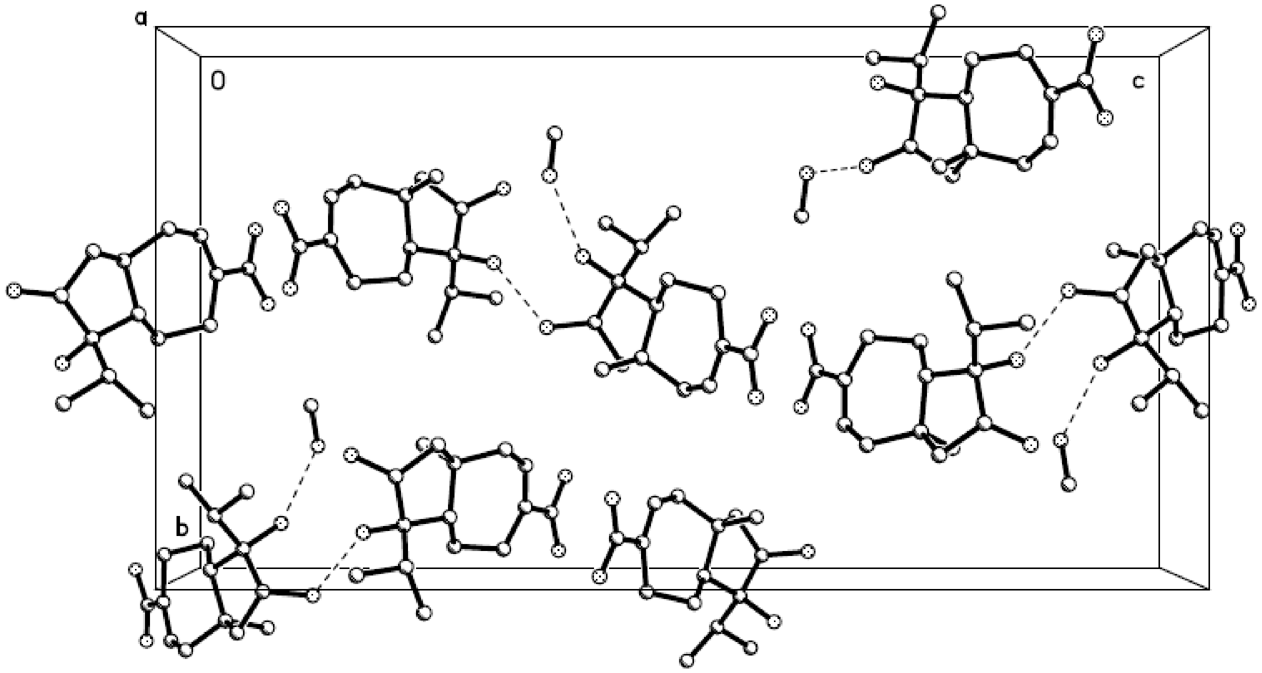

Supplement: Supplementary file 1 [file antibiotics-10-00213-s001.pdf]
